# Organically-preserved multicellular eukaryote from the early Ediacaran Nyborg Formation, Arctic Norway

**DOI:** 10.1038/s41598-019-50650-x

**Published:** 2019-10-10

**Authors:** Heda Agić, Anette E. S. Högström, Małgorzata Moczydłowska, Sören Jensen, Teodoro Palacios, Guido Meinhold, Jan Ove R. Ebbestad, Wendy L. Taylor, Magne Høyberget

**Affiliations:** 1 Department of Earth Science, University of California Santa Barbara, Santa Barbara, CA 93106 USA; 2 Department of Earth Sciences, Uppsala University, 752 36 Uppsala, Sweden; 3 Arctic University Museum of Norway, UiT - The Arctic University of Norway, N-9037 Tromsø, Norway; 4 Área de Paleontología, Universidad de Extremadura, E-06006 Badajoz, Spain; 5 School of Geography, Geology and the Environment, Keele University, Keele, ST5 5BG UK; 6 Department of Sedimentology and Environmental Geology, University of Göttingen, Goldschmidtstraße 3, 37077 Göttingen, Germany; 7 Museum of Evolution, Uppsala University, 752 36 Uppsala, Sweden; 8 Department of Geological Sciences, University of Cape Town, Rondebosch, 7701 South Africa; 9 Rennesveien 14, N-4513 Mandal, Norway

**Keywords:** Palaeontology, Palaeoecology

## Abstract

Eukaryotic multicellularity originated in the Mesoproterozoic Era and evolved multiple times since, yet early multicellular fossils are scarce until the terminal Neoproterozoic and often restricted to cases of exceptional preservation. Here we describe unusual organically-preserved fossils from mudrocks, that provide support for the presence of organisms with differentiated cells (potentially an epithelial layer) in the late Neoproterozoic. *Cyathinema digermulense* gen. et sp. nov. from the Nyborg Formation, Vestertana Group, Digermulen Peninsula in Arctic Norway, is a new carbonaceous organ-taxon which consists of stacked tubes with cup-shaped ends. It represents parts of a larger organism (multicellular eukaryote or a colony), likely with greater preservation potential than its other elements. Arrangement of open-ended tubes invites comparison with cells of an epithelial layer present in a variety of eukaryotic clades. This tissue may have benefitted the organism in: avoiding overgrowth, limiting fouling, reproduction, or water filtration. *C*. *digermulense* shares characteristics with extant and fossil groups including red algae and their fossils, demosponge larvae and putative sponge fossils, colonial protists, and nematophytes. Regardless of its precise affinity, *C*. *digermulense* was a complex and likely benthic marine eukaryote exhibiting cellular differentiation, and a rare occurrence of early multicellularity outside of Konservat-Lagerstätten.

## Introduction

The late Neoproterozoic interval was a critical time of turbulent environmental changes and key evolutionary innovations. Postdating global, low-latitude glaciations^1^ and coincident with progressive oxygenation of the oceans^2^ that intensified in the Ediacaran Period^3^ was the emergence of complex ecosystems^4–7^. Diversification of multicellular life across various eukaryotic groups included macroscopic algae^8–10^, soft-bodied Ediacara-type biota^6,11^, biomineralizing and vermiform organisms^12,13^. Although multicellularity evolved multiple times amongst Precambrian eukaryotes^14,15^, it only rose to prominence in the late Proterozoic^16^ and its Neoproterozoic record is dominated by taxonomically problematic groups, often restricted to cases of remarkable preservation^9,16–19^ (with rare exceptions^20^). The extent to which early multicellular fossils occur due to special taphonomic conditions rather than a true diversification is unclear (cf.^21^), so data outside of cases of exceptional preservation are of special value. The macroscopic Ediacara-type biota appears shortly after a widespread negative carbon isotope excursion^22^ and a short-lived low-latitude glaciation (Gaskiers^23^), but little is known about Earth’s biosphere during the pre-glacial interval of the earliest Ediacaran outside of Konservat-Lagerstätten^17,22,24^. The early Ediacaran fossil record includes microfossils (Doushantuo-Pertatataka-type acritarchs^25,26^) and carbonaceous compression fossils identified as algae or problematica^9,10,22^.

**Figure 1:**
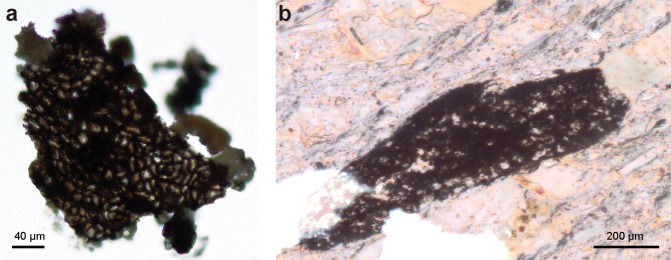
Transmitted light photomicrographs of *Cyathinema digermulense* gen. et sp. nov. from the Nyborg Formation. (**a**) Fossil tissue extracted via palynological maceration, TSGf18422 (England finder position: U38). (**b**) Carbonaceous fossil embedded in shale in thin section, TSGf18424 (J61/1).

**Figure 2:**
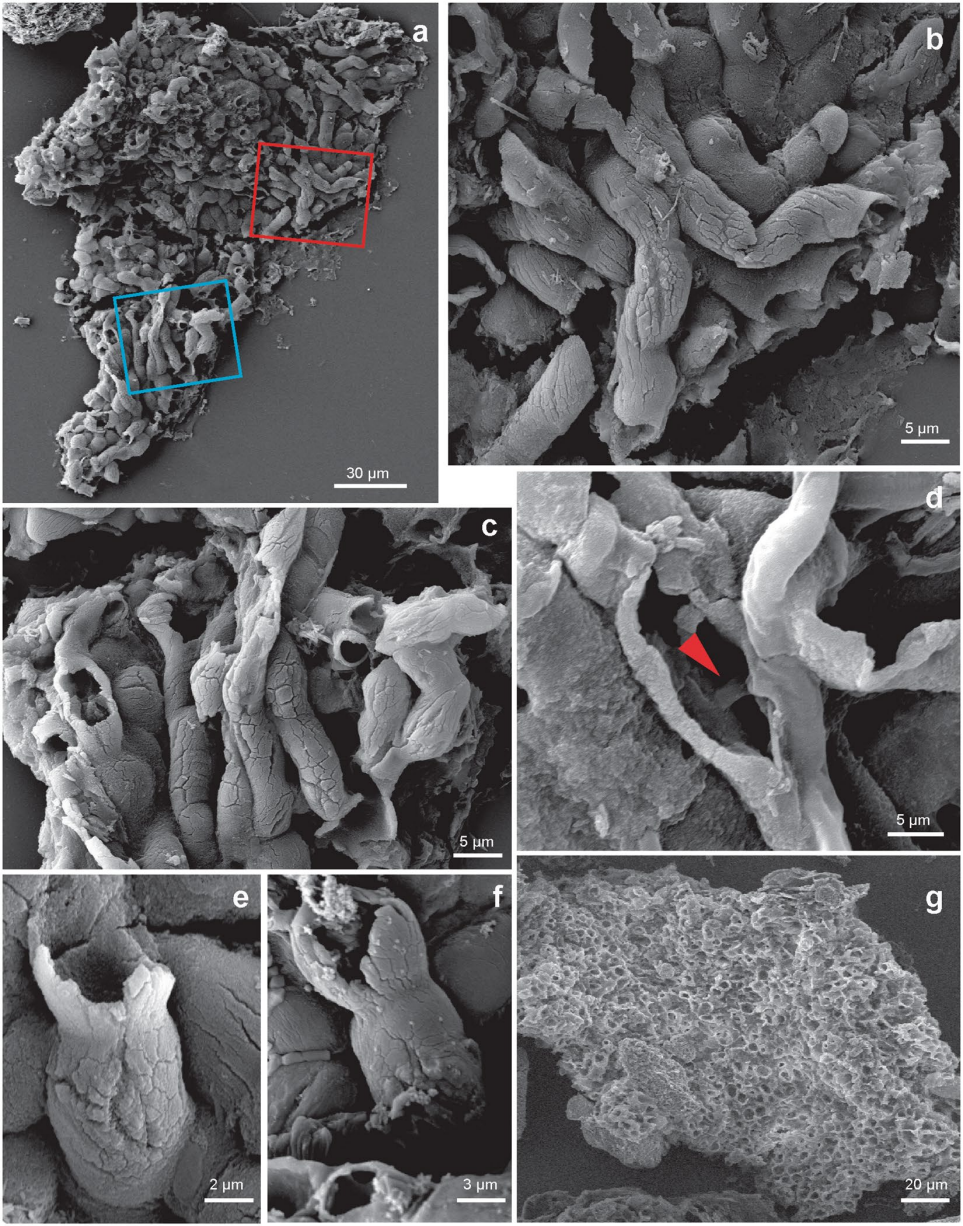
Tubes composing the sheet of *Cyathinema digermulense* gen. et sp. nov. (**a**–**c**) A fragment of *Cyathinema*, stacked tubes are visible on one side. TSGf18420d (U40/2). Boxes in (**a**) indicate where the magnified elements illustrated in figures (**b**) in red and (**c**) in blue are located. (**d**) A fractured tube where with a visible interior. A narrower siphon-like structure is running perpendicular to the tube walls (red arrow). Only one broken specimen shows tube interior with a “siphon”, but it is unclear if that is a true character. No internal features are observed in other specimens. TSGF18421. (**e**) TSGf18420e. (**f**) TSGf18423b. (**g**) Top-down view of the apical side, TSGf18423a (uncoated sample for EDX analysis). All images are scanning secondary electron (SEM) photomicrographs.

**Figure 3:**
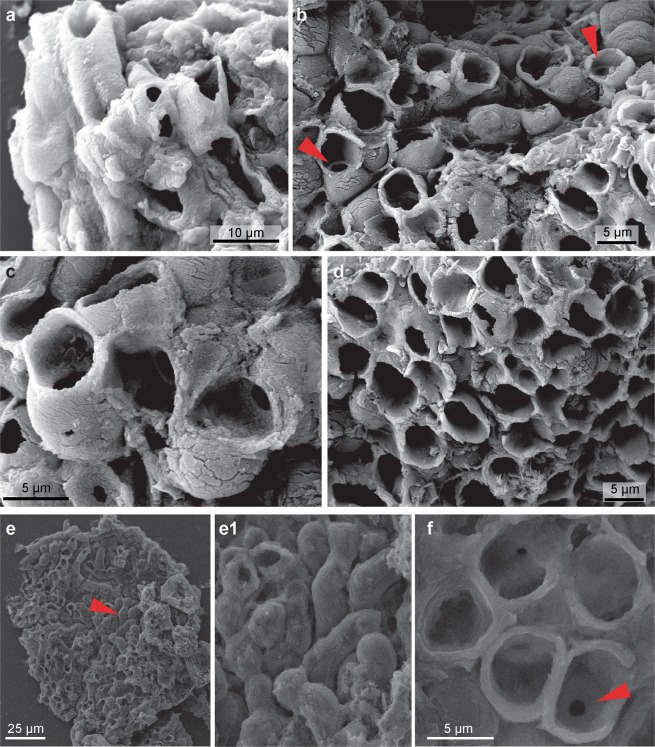
Cup-shaped terminations on the apical side of *Cyathinema digermulense* gen. et sp. nov. (**a**) TSGf18420b (N39). (**b**–**d**) TSGf18423c. (**b**) Circular inner openings (red arrow) are connecting the cup-shaped termini with cells underneath. Occasional unopened domed termini are seen on the right. (**e**) TSGf18423b. (**f**) TSGf18423a, arrow indicates inner opening inside the cup-like terminus. (**e–f**) Uncoated samples in environmental low vacuum mode for EDX analysis. Well-preserved cups have an even and well-defined outline (**f**), although they commonly appear torn (**d**) which may be a result of sample processing. All images are SEM photomicrographs.

Here we describe a new organically-preserved multicellular (or colonial) eukaryote *Cyathinema digermulense* gen. et sp. nov. (Figs 1–3) from the intraglacial Nyborg Formation (c. 635–580 Ma) on the Digermulen Peninsula, Arctic Norway. Fragmentary specimens are a common component of the upper Nyborg assemblage. Scanning electron microscopy (SEM) revealed an unusual arrangement of hollow tubes terminating in cup-shaped structures previously not recorded among Proterozoic microfossils, that justify the establishment of a new taxon. *C*. *digermulense* resembles epithalli of red algae otherwise known from preservation in phosphorites, and also shares characteristics with other protists and putative Ediacaran sponge fossils (Fig. S5).

## Results

### Geological setting

The Nyborg Formation, lower Vestertana Group assigned to the early Ediacaran^27,28^, occurs between two glaciogenic diamictites (Smalfjorden and Mortensnes formations), exposed in Finnmark, Arctic Norway (Fig. S1). It consists of interbedded purple sandstones and shale-and-siltstone packages, with cap dolomites at the base associated with the Marinoan glaciation^28,29^. Nyborg Formation sediments represent a shallowing-upwards sequence, and tidally-influenced marine environment^28,30^. The Mortensnes diamictite is overlain by siliciclastics of the Stáhpogieddi Formation, which contain Ediacara-type fossils and ichnofossils^31–33^. Previously reported organic-walled microfossils from the Nyborg Formation include leiosphaerids and ‘*Trachysphaeridium*’^34^.

### Systematic palaeontology

Domain EUKARYA Woese *et al*. 1990.

 Genus *Cyathinema* nov.

### Type species

*Cyathinema digermulense* gen. et sp. nov. (Figs 1–3) from the lower Ediacaran Nyborg Formation, Finnmark, Norway.

### Etymology

From Latin *cyathus*, meaning a cup/goblet, and *nema*, a thread; referring to cup-shaped terminations of tubular threads that comprise the fossil.

### Diagnosis

As for type species.

*Cyathinema digermulense* sp. nov.

### Etymology

In reference to the Digermulen Peninsula, where the fossils were first discovered.

### Holotype

TSGf18420c (O37/2) (Fig. 2a–d), from the Nyborg Formation, 20 cm below the Mortensnes Formation, catalogued in the palaeontological collection of the Arctic University Museum of Norway.

### Material

24 specimens from the uppermost Nyborg Formation, samples D14-N1, D14-N2, D16-HA-69, D16-HA-74.

### Locality and stratigraphy

Siltstones and claystones of the uppermost Nyborg Formation, Vestertana Group, exposed on the southeastern shore of Digermulen Peninsula in Finnmark County, Arctic Norway (N70°34.005′, E028°06.739′, Fig. S1), 5 cm to 8 m below the Mortensnes Formation diamictite correlated^28^ with the Ediacaran Gaskiers glaciation.

### Diagnosis

Thalloid, sheet-like body with cellular assortment, comprising a meshwork of interwoven or stacked tubules that terminate in dome-like or cup-shaped structures. Sheets reticulate in appearance on one side. Tubular elements hollow, non-branching, variable in length. Tube extremities commonly open-ended and cup-shaped, occasionally closed distally. Cups and tubes separated by a constriction with a small, circular opening. Inner opening within cup-shaped terminus extends into the hollow tube, and varies in size up to 30% of the cup diameter. Where the cup is not visible, the internal constriction (with inner opening) is still clear. Wide, short neck develops occasionally between tubes and cup-termini. Carbonaceous walls robust and taphonomically recalcitrant.

### Description of fossils

The new Nyborg microfossils are carbonaceous, rectangular to cigar-shaped, sheet-like structures, and they vary in size, shape, and number of individual elements (tubes). The fossils extracted from the rock matrix via palynological acid maceration appear as semiopaque, dark-brown structures when observed via transmitted light microscopy (Fig. 1a). Colour indicates moderate thermal alteration of the organic matter (TAI = 3.5–4), which is corroborated by Raman spectroscopy (Fig. S4). All recovered fossils occur in fragments (up to 327 µm long, mean ($\bar{{\rm{x}}}$) = 158 µm, standard deviation (st.dev.) = 78.15, number of measured specimens (n) = 14) as thin, flattened sheets (c. 10 µm thickness) (Table S1). Cup-like structures on specimens’ surface give the sheets a reticular appearance (Fig. 1).

**Figure 4:**
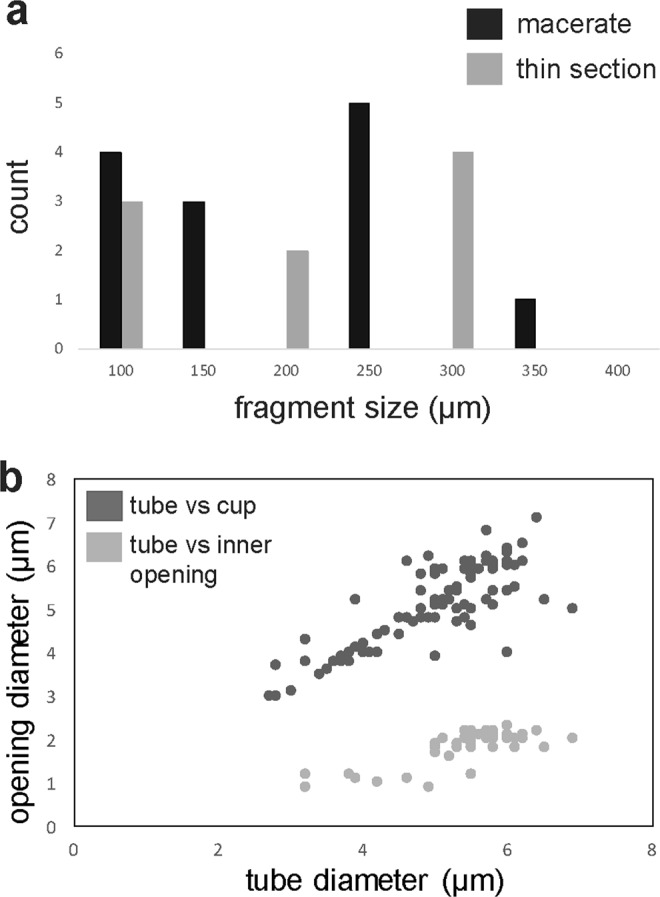
Measured dimensions of the fossils and elements of *Cyathinema digermulense* gen. et sp. nov. (**a**) Size distribution of fossil fragments extracted via acid maceration (black) and fragments observed in thin sections (grey). (**b**) Crossplot showing tube diameter plotted against cup diameter (dark grey, n = 92) and tube diameter vs. inner opening diameter (light grey, n = 42). The diameter of the cup structure follows the width of the tubes, but the side of inner openings is largely consistent, even if it occurs in larger tubes. Only tubes on which the large opening (cup) diameter and the inner opening were visible were measured and compared on the crossplot.

Anatomical details are more recognizable via high-magnification SEM. Fossils comprise a pattern of densely stacked and occasionally interwoven tubes (Figs 2a,b and 3e) crowned with either domed termini (Fig. 3b) or straight or cup-like openings (Figs 2e–f and 3b–f). Tubes are on average 5.02 µm wide (st.dev. = 0.94, n = 91), most commonly parallel, occasionally intertwining, but never branching. Tubes are mostly concealed along their lengths. On specimens where their entire length could be measured, tubes range from 7–22.5 µm ($\bar{{\rm{x}}}$ = 15.5 µm, st.dev. = 5.4 μm, n = 38) (Table S1). Individual tubes are hollow, terminate in closed domes or open cup-shaped structures (Figs 2d–f and 6) and connect to the interior of the “cup” through a small, circular opening (Fig. 3b,c,f). The inner opening is consistent in size up to 1/3 of the overall cup size ($\bar{{\rm{x}}}$ = 1.6 μm, st.dev. = 0.52 μm, n = 96), has a well-defined outline, so it likely represents a true character. The average diameter of the cup-structure is 5.05 μm (st.dev. = 1.1 μm, n = 195), and it corresponds to the tube diameter (Figs 4b and S3). The tubes narrow before terminating in the wider cup-structure, thereby creating a neck-like feature seen in some specimens (Fig. 2e–f). Cup-like structures often have an uneven margin and appear broken (Fig. 3d) and could have been formed by the breakage of the tubes, but well-preserved specimens possess cups with a well-defined, smooth margin (Fig. 3f). Tubes are robust (Figs 2e and 3f), 0.4–1.1 μm in wall thickness, and optically dense. Cups—tube terminations with an inner opening (Fig. 3b,c), have been observed only on one side. When visible, the opposite side of a tube may be open-ended due to breakage, but does not possess a cup-like termination segregated from the tube via small, inner opening. Following those observations, it appears true apical openings (cups with inner openings) are distributed only on one side of the fossil. Cups and the closed domed apices share the same diameter (Fig. 3b), though closed tips are rarer and randomly distributed on the surface of the fossil, meaning that the cups likely formed by opening or breakage of domed apices, and could have functioned as apertures. It is unlikely that these structures are a result of inward collapse because high-magnification SEM reveals structures within the cups, such as inner opening (Fig. 3b,c,f) extending into a hollow tube. There are few fractured specimens where the tube interior is visible; a narrow siphon is observed in one specimen (Fig. 2d), but others do not reveal additional internal features.

Although the tubes and cup-like termini vary in size, they are uniform in diameter on a particular fragment (Table S1; Fig. S2). Fossils with larger tubes are associated with larger cups and internal openings (Fig. 4b). The fossil appears stratified; cups with inner openings only occur on one end of examined specimens, presumably the fossil surface. In addition to the material extracted from the rock matrix, *Cyathinema* was also observed in thin sections (Fig. 1b), which rules out the likelihood of originating from recent contamination. Specimens in thin sections are usually bigger than the macerated material (Fig. 4a, Table S1) and commonly elongate. Size ranges from 68 to 413 μm ($\bar{{\rm{x}}}$ = 231.7 μm, st.dev. = 120.3 μm, n = 10). Breakage and tearing visible around the edges (Fig. 1b) suggest that all specimens are allochthonous and were not deposited complete or preserved in life position. This is further supported by the orientation of fossil fragments’ long axis at various angles to the laminar bedding, which indicates that the fossils underwent transport and re-deposition like clasts. As a result, the full size and morphology of *Cyathinema* remain unclear.

### Chemical composition

**Figure 5:**
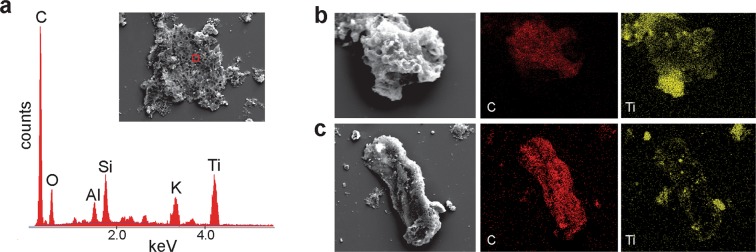
Elemental composition of macerated fossils from energy dispersive X-ray microanalysis. (**a**) Point-analysis on a tube of *Cyathinema digermulense* gen. et sp. nov. shows that the fossil is primarily composed of carbon. TSGf18420a. (**b**,**c**) Elemental mapping shows the two most abundant elements. The fossils are composed of carbonaceous matter, and often covered with debris of titanium, likely originating from rutile. (**b**) TSGf18424. (**c**) Specimen of a rolled-up sheet, TSGf18420b.

Energy-dispersive X-ray microanalysis (EDX) and Raman spectroscopy determined that the meshwork microfossils of the Nyborg Formation are composed mainly of carbonaceous material (Fig. 5). Elemental mapping showed that the surface of some macerated specimens is lightly coated with aluminosilicates and most abundantly titanium oxides derived from the debris of the host sediment (Fig. 5b,c). Raman spectra collected from the wall of *Cyathinema* show two prominent vibrational peaks at ~1367 cm^−1^ and 1621 cm^−1^ representing disordered (D) and graphitic (G) bands of organic carbon (Fig. S4). Intense D-band indicates low epizonal thermal maturity of the carbonaceous material^35^.

### Preservation

The fossils are preserved as organic matter in shale and siltstone. Raman spectrometry showed moderate thermal maturity of carbonaceous matter (Fig. S4). The fossils exhibit some degree of degradation, but usually have a well-preserved, featureless surface (Fig. 3). Good organic preservation and the ability to withstand acetolysis indicate that the original tissue was probably composed of recalcitrant material like organic-walled microfossils and small carbonaceous fossils, and/or deposited under conditions inhibiting biodegradation (cf.^36^). Both in the macerated material and thin sections, *Cyathinema* specimens are truncated, which can be attributed to short transport after death rather than sample processing.

### Remarks

Incompleteness of all recovered specimens implies they were a part of a larger structure, perhaps with higher preservation potential than the rest of the organism. Cellular differentiation (elongate tubular cell, and rounded terminal cell connected to the tube via small opening) also supports a multicellular nature of *Cyathinema*. Even though they represent a single component of a larger entity, the Nyborg fossils share features with red algae (Rhodophyta)^8,37,38^, colonial protists^39^, demosponge larvae or stolon cells (Porifera)^40–42^ and elements of *Eocyathispongia*^43^ (Table S2; Fig. S5).

## Discussion

### Function and reconstruction

All recovered specimens of *Cyathinema* are redeposited fragments, so the original overall shape and size of the fossils are yet unknown. This is unsurprising considering that small organisms or disarticulated body parts are more predisposed to transport. The sheets were a part of a larger entity and may be classified as an organ-taxon, i.e. disassociated body-parts whose contributing organism is not preserved, or unknown^44^. No other sheet morphologies were recovered from the Nyborg Formation, including previous micropalaeontological studies^34^. One explanation could be higher preservation potential of the tubes that form *Cyathinema*. Fossils were able to withstand palynological acid extraction, as is common among recalcitrant material preserved organically within shales^36^. This innate recalcitrance suggests that cups-and-tubes were a form of cuticular material or that they contained cell walls. Other modes of fossilization, e.g. early silicification^45^ or phosphatization^37^ that allow exceptional preservation of more delicate elements could potentially record other components of this organism.

**Figure 6:**
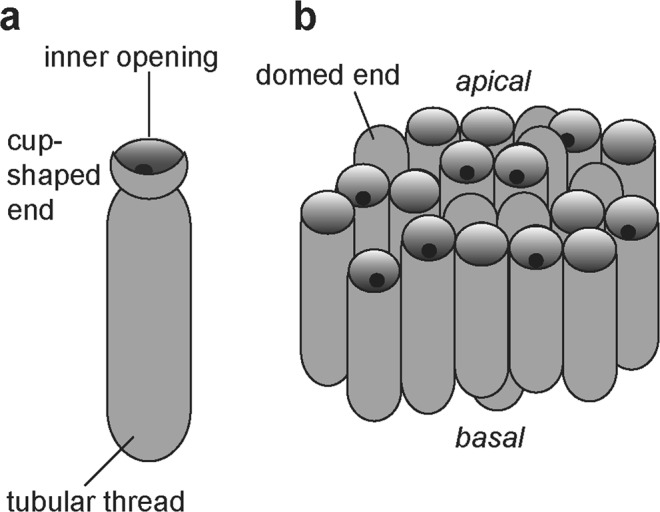
Schematic illustration of a set of stacked tubes of *Cyathinema digermulense* gen. et sp. nov. (**a**) individual tube terminating in a cup-shaped structure. A small, round opening inside the cup connects to the interior of the tube. (**b**) A stack of tubes of varying lengths. The “cups” and the closed, domed termini usually occur on the apical side of the fossil. This arrangement represents a single layer, in a way the fossils usually occur. Rare tubes that are distally closed are randomly distributed.

Disassociated fossils are difficult to classify, yet they offer glimpses into the fossil organism’s function and thus palaeobiology, keeping in mind the caveat of convergent evolution. *Cyathinema* appears polarized along the basal-apical axis (Fig. 6). Comparable tissues in various organisms, from epithallus of red algae to chambered walls of demosponges, possess tubes with openings or cups on the external layer of the organism^37,42,43^. We therefore assume that cups represent the organism’s external layer. Cups and rarer domed termini are likely different stages of the same structure. The tube-and-cup arrangement of *Cyathinema* and its dimensions are analogous to epithelial thalli common in algae and fungi, as well as basal plants; or rhizoids–protuberances from algal epithelial cells that serve as an anchoring and a nutrient uptake structure^46,47^. Red algal epithallus (Fig. S5c) is produced and shed throughout life to limit fouling or prevent other organisms from attaching to the alga and overgrowing it^48^, or as a coping strategy for grazing pressure^49^. Alternatively, noting that the fossilised tissue was more robust and preservable than the rest of the organism, and thus presumably costlier to produce, it may represent a reproductive structure. The tube-and-cup arrangement is also present in apical/external reproductive cells of red algae^38,50,51^.

Inner opening connected into tube underneath a cup (Fig. 2b,c,f) resembles flagellum attachment points in epithelia of demosponge larvae^40,42,52^. The flagella are commonly used in phagocytosis^53^. But these features would have been easily destroyed due to low preservation potential, and are not present in red algae.

Regardless of the exact phylogenetic affinity of the Nyborg Formation fossils, their epithallus-like anatomy and fragmentary nature indicate they were an outer layer of a larger, multicellular eukaryotic organism, that played a role either in nutrient-uptake, protection or epithelial sloughing, reproduction, or a combination thereof. The geological context shows that fossils were deposited in a shallow-slope marine environment^28,30^, in nutrient-limited oceans (cf.^1^). The source organism was probably a benthic, marine eukaryote. The assumption that *Cyathinema* represents elements of a meso- or macroscopic organism is plausible; diverse multicellular eukaryotes had already evolved by the pre-Gaskiers interval of the early Ediacaran, as evidenced by macroscopic carbonaceous compressions from the Lantian Formation^9^ and suggested metazoan embryos and/or multicellular protists from Weng’an phosphorites^19,54^.

### Comparison with fossil and extant organisms and implications for phylogenetic affinity

The morphology of *Cyathinema* stands out from previously described Proterozoic taxa of macerated fossils, in terms of overall construction, individual elements, and preservation. The interpretation of biological affinity is challenging due to a lack of complete specimens. We rely on well-preserved, fine-scale anatomical details revealed by SEM to assess similarities with extinct and extant clades.

Fragments of flattened cellular sheets from the Mesoproterozoic Gaoyuzhuang Formation (Fig. 7b–e^55^) resemble *Cyathinema* in transmitted light microscopy. They were found in association with cuneate carbonaceous compression fossils and interpreted as disarticulated remains of those macroscopic forms^55^. Unlike *Cyathinema*, Gaoyuzhang fragments possess irregular and cuspate cells and lack tubes.

*Cyathinema* also resembles charcoalified body parts of the fungus *Prototaxites* sp. from the Silurian–Devonian transition of Welsh Borderland (Fig. 6a–c^56^). The hymenial layer of *Prototaxites* consists of a reticulate epihymenium and tubular threads resembling the cups-and-tubes in *Cyathinema* (Fig. 2a), although the hymenial threads in *Prototaxites* are usually considerably longer (>200 μm). Among living fungi, hymenium is present in Basidiomycota and Ascomycota and serves as a surface layer of differentiated tubular cells (Fig. S5) from which reproductive structures are developed^57^. Unlike the terrestrial *Prototaxites*, *Cyathinema* probably inhabited a shallow-marine environment^28,30^, but due to transport we cannot exclude an origin in a deltaic environment. Apart from a putative occurrence of lichens in the Doushantuo Formation^58^ which cannot be confidently distinguished from co-occurring thalli of red algae, there is little evidence for fungal fossils in the Ediacaran. Fungi are thought to have evolved and expanded in the Ordovician–Silurian^56,59,60^ (although see^61^), but the divergence within the Opisthokonta (Holozoa including the animals and Holomycota including the fungi) must have occurred by the late Neoproterozoic due to the presence of animals (cf.^15,62^), so fossils of the total-group Holomycota would not be unexpected.

Charcoalified organ-taxa of *Prototaxites* are a part of an extinct polyphyletic group of complex, organically-preserved, tubular and reticular sheets that contain disarticulated fragments of larger organisms: nematophytes^63^, which bear further similarity to *Cyathinema*. Various nematophyte morphotypes have been interpreted as: seaweeds/red algae^64^, early embryophytes^65^, and fragments of a large fungus^56^. Traditionally, Nematophyta included land-dwellers, however fossils from marine settings have been reported from the Silurian of Gotland, Sweden^64^. *Nematothallus* (Figs 2a–j, 5f, 5n^65^) and *Nematothallopsis* (Fig. 5a–c^64^) common in Silurian–Devonian strata, resemble *Cyathinema* tubes in shape and size. Although the thalli of Silurian nematophytes lack cup-like structures, they possess a reticulate cuticle (Fig. 1a–c^64^, Fig. 1a–b^65^), and cells with dome-shaped tips^65^ similar to some Nyborg specimens (Fig. 2b). Unlike morphologically variable nematophytes^63^, all specimens of *Cyathinema* share the same form and the Nyborg Formation hosts no evidence of differentiated organ-taxa akin to Silurian–Devonian forms. Nevertheless, *Cyathinema* is the oldest fossil exhibiting organization present among some nematophytes. It is possible that the source organism contained only one body-part type with high enough preservation potential to leave a fossil record, unlike younger Palaeozoic nematophytes.

Tubular thecae ending in flagellar chambers are present in colonial choanoflagellates–flagellated heterotrophic protists and sister-group to Metazoa^66,67^. Colonies of modern non-loricate forms consist of clumps of collared cells (chart 9^39^, Fig. 1.6^68^), or branched tubes^39,52^. Inside the collar is a small round opening (Fig. 5^52^) where the flagellum is attached, similar to the inner opening of *Cyathinema* (Fig. 3b–c,f). The cups are made of the same recalcitrant material as the tubes and are thus not homologous with the collars of modern choanoflagellates which consist of dense microvilli or tentacles and a mucus sheath^67,68^ with low preservation potential. But the inner opening could have played a similar feeding role as in modern tubular flagellated protists. Accepting the estimates for the appearance of total-group animals and choanoflagellates by late Neoproterozoic^62,66^, choanoflagellates or a related colonial protistan clade are not unexpected in the Ediacaran. Although it should be noted that their fossil record is virtually non-existent, even that of silica-bearing loricate forms^15,67^.

Reproductive structures (sporangia) of some colonial slime molds (Mesomycetozoea, Opisthokonta) also consist of clusters of stacked tubes with domed tips, e.g. *Stemonitis* (Fig. S5i,j), *Metatrichia* (Fig. 121a,b^69^). Upon spore release, tube tips become cup-like cavities^69^ that resemble *Cyathinema*, but are much larger (Fig. S5i,j). Sporangial clusters of *Trichia favoginea* (Fig. 104^69^) contain uneven ruptures similar to some poorly preserved specimens of *Cyathinema*. Akin to choanoflagellates however, the fossil record of slime molds is extremely poor and restricted to cases of exceptional preservation in amber^70^.

Among protists, *Cyathinema* also resembles deep-sea foraminifer *Syringammina fragilissima* (Xenophyophorea), one of the largest unicellular organisms growing up to 20 cm which exhibits an arrangement of open-ended tubes, but it is an order of magnitude larger than *Cyathinema*. *Syringammina* also has openings all over the fossil^71^, whereas *Cyathinema* cup-structures with inner openings are one-sided. Possible xenophyophore fossils are known from younger Ediacaran units^33,72^, but only agglutinating forms. *Cyathinema*’s carbonaceous wall and regular arrangement of tubes render the xenophyophore affinity unlikely.

*Cyathinema* also shares morphology with elements of phosphatised fossil *Eocyathispongia qiania* from the Doushantuo Formation in China^43^, interpreted as the oldest sponge-grade body fossil. Chamber walls of *Eocyathispongia* consists of stacked, hollow tubes with occasionally open ends (Figs 4b–c, 5d–e^43^) that correspond in shape and size to *Cyathinema* tubes (5–8 μm wide). The authors compared cellular-level characters in *Eocyathispongia* to demosponge papillae used for nutrient uptake, or exhalent pores through which the choanocytes filter water^43^. *Eocyathispongia* is a complex taxon with unusual macro-morphology and few poriferan synapomorphies. Its demosponge affinity was recently questioned^73^ on the basis of minute overall size and insufficient available surface area for efficient filter-feeding. Although it is possible that such a small sponge could have also depended on protistan and bacterial (chemo- or photoautotrophic) symbionts for nutrients, the evidence for that relationship has not yet been found in these fossils. Among other stem-group and early sponge fossils^73^ most taxa seldom preserve cellular structures for direct comparison. Molecular clock estimates predict a Cryogenian origin of the Porifera^62,74^, which is supported by the biomarker record of steranes likely produced by demosponges^75,76^. Sponge body fossils on the other hand, are conspicuously absent from the Cryogenian–Ediacaran interval, and most of the poriferan fossil record in the Precambrian is problematic^77,78^. This missing record may be explained by a late invention of metazoan biomineralization, which is in agreement with demosponge-specific biomarkers in the Cryogenian^75,76^ (although see^79^). Demosponges can produce siliceous spicules, but many are soft-bodied (composed of collagen spongin or chitin^80^) and would have thus been less likely to leave a fossil record. The earliest possible biomineralizing/encrusting poriferan appeared in the terminal Ediacaran^13^. Regardless of its affinity, the external layer of cells in *Eocyathispongia* (Fig. 2a^43^) strongly resembles *Cyathinema*. The two fossils may be related, or represent the same problematicum, where each is preserved differently.

Additional analogous structures are present in modern Porifera. Arrangement of *Cyathinema* sheets is similar to the epithelial layer of extant demosponge larvae (Fig. S5h), e.g. *Sycon coactum*, *Ircinia oros*, and *Halisarca dujardini* (Figs 3.34a, 29a–b^40^, Fig. 1d^42^, Figs 3, 4a^52^). Tubes further resemble elongate inner cells of the stolon in an extremophilic demosponge *Amphilectus lobatus* (Fig. 6c,d^41^). Stolons are outgrowths produced by some species of Demospongiae, allowing them to crawl along the substrate^41^. A stolon-like function would explain the incompleteness of *Cyathinema*, yet there is no indication of the fossilization potential of stolon cells. Based on morphological comparison, *Cyathinema* may represent the epithelial layer or a stolon of a stem-group soft-bodied sponge, but until a complete specimen is found, affinity to other organisms with epithelium cannot be excluded.

Ostensibly, simple columnar epithelium in animals (Figs S5f,g) and the epithelial layer of *Trichoplax adherens* (Placozoa) also resemble the new fossils. Epithelial cells in cross-section (Fig. 3d^81^) correspond in shape and size to *Cyathinema* tubes, and terminate in concave structures. The appearance of cells between cross-section and three-dimensionally preserved microfossils may vary, but the apices of dorsal epithelial cells of *Trichoplax* (Fig. 1b^81^) are also similar to the reticulate appearance of *Cyathinema* under a transmitted light microscope. However, animal cells lack a wall that would facilitate their organic preservation.

Most characteristics of *Cyathinema* are shared by clades within multicellular red algae (Rhodophyta). Comparable taxa from the lower Ediacaran Doushantuo Formation in China, which hosts numerous permineralized, pseudoparenchymatous multicellular organisms, are considerably larger than the Nyborg fossils, but possess an external layer of elongate cells ending in domed structures (Figs 4–5, 26, 41–42^8^, Fig. 3c,d^37^, Fig. 3.3^82^) that correspond in size and shape to *Cyathinema*. Arrangement of tubes terminating in “cups” giving the fossils a reticular appearance on one side is similar to the external layer of *Wengania globosa*^8,82^ and *Thallophyca corrugata*^83^, both of which have been assigned to Florideophyceae. The epithalloid layer of Doushantuo fossils was compared to the reproductive layer (spermatangia) present in modern florideophytes, e.g. *Gracilaria*^37^. Phylogenomic data and molecular clocks estimate the origin of Florideophyceae in the early Tonian^84^, so the presence of florideophyte algae in the Vestertana Group would be in concert with their antiquity. Younger fossil^85^ and modern red algae^38^, especially amongst order Corallinales (Fig. S5c,d; Fig. 6I–L^86^), also exhibit a thalloid construction with tubular cells in the external layer. Ancestral corallines and many younger red algae are crustose organisms, unlike *Cyathinema* which was originally soft-bodied, but the simple body plan of an external layer appears conserved across Florideophyceae. The inner opening of *Cyathinema* (Fig. 3f) is comparable to pit connections of red algae–holes between the walls of elongate cells, infilled by proteinaceous “pit plugs”^87,88^. Epithallial cells or their pits on the surface of *Phymatolithon rugulosum* (Fig. 4b–d^88^) contain an inner opening and their top cell wall may be intact or broken out, similar to *Cyathinema* cups and domed termini (Fig. 3b). Diploid multicellular stage (sporophyte) of the nemalialean alga *Titanophycus validus* (Florideophyceae) consists of tubular filaments terminating in dome-like gonimoblasts (Fig. 2.2^38^) corresponding in size to *Cyathinema* tubes. Comparable construction of an outer layer fitting the dimensions of *Cyathinema* tubes, is also common in pores of the conceptacle (reproductive layer) of *Lithothamnion steneckii* (Figs 3a–d, 4a^51^,) and *Osmundea spectabilis*^89^. Interestingly, *Osmundea*’s epithallus produces trichoblast cells surrounded by a robust, bi-layered wall^89^. Its mucilage sacs contribute the material for wall-formation; a process which may promote successful organic preservation in shales, and thus could explain the preservation of *Cyathinema* outside exceptional circumstances. Also in agreement with red algal construction is the lumpy appearance of some tubes (Fig. 2b,d), which indicates they may have hosted multiple cells inside.

The oldest red algal fossil is *Bangiomorpha pubecens*^90^ from cherts of the Angmaat Formation, Canada, recently constrained to ~1048 Ma by rhenium–osmium geochronology^91^. Red algae possibly originated around 1.6 Ga, accepting an occurrence in the lower Vindhyan Supergroup, India^92^ (of questioned age^91,93^). Despite the group’s longevity, the Neoproterozoic record of red algae is sparse; they are known mainly from phosphorites of the Ediacaran Doushantuo Formation, China^8,37^, with a single possible occurrence in the Cryogenian Taishir Formation, Mongolia^94^, while none were extracted from shales or siltstones. If red algal affinity is accepted for *Cyathinema*, the occurrence of their epithalli within shales demonstrates the feasibility for red algae to become preserved organically.

In sum, *Cyathinema* shares features with various clades, however due to the low preservation potential of choanoflagellates and slime molds, and its fragmented state, it is more likely that it represents a part of a larger entity, such as organisms with an epithelial layer (Fig. S5). A differentiated outer cell layer has evolved multiple times in various multicellular eukaryotes: red algae (called epithallus), brown algae (epidermis), metazoans (epithelium), fungi (hymenium/epidermis) (Fig. S7). The structure is likely polyphyletic, and its phylogenetic distribution among eukaryotes is little understood. Epithelial layer *sensu lato* is an organism’s outer layer of often tube-shaped cells (Fig. S5), used in overgrowth prevention, reproduction, gas exchange, or morphogenesis^48,95^. With a long Precambrian record and most shared characters, red algae are the most likely candidate for the affinity of *Cyathinema*, yet relationship to other epithelium-bearing organisms cannot be excluded. Regardless of its affinity, *Cyathinema* provides support for the presence of epithelial organisation by the late Neoproterozoic. An external layer of differentiated cells (epithallus/epithelium/epidermis) is present in various multicellular organisms (Fig. S7), and has been crucial for their diversification and expansion^96^. A fossil record of epithalli/epithelia may narrow down the timing of the establishment of cell polarity^96^.

## Conclusions

The fossiliferous late Neoproterozoic sedimentary succession on the Digermulen Peninsula provides an opportunity to investigate the biosphere between turbulent end-Neoproterozoic glaciations. Unique carbonaceous fossils *Cyathinema digermulense* sp. et gen. nov. were recovered from the Nyborg Formation and represent remains of a multicellular eukaryote. It is characterised by stacked tubes ending in cup-like structures (Figs 2–3). The fossils are carbonaceous and preserved organically (Fig. 5). Cellular differentiation, fragmented state due to transport before burial, indicate the fossils were likely parts of a larger organism, and had a higher preservation potential than the rest of its components. The Nyborg fossils exhibit features of an external cell layer (epithelium/epithallus). Such a tissue would have benefitted the organism in a variety of ways: (1) avoidance of overgrowth or sedimentary disturbance, (2) fouling limitation, (3) reproduction, or (4) phagocytosis/filtration. Signs of cellular differentiation and epithalloid arrangement of *Cyathinema* suggest that the source organism was a complex and probably epibenthic eukaryote. *Cyathinema* resembles various extant and fossil groups: modern red algae^38,48,88^ and their fossils^8,37^, nematophytes^64^, putative Ediacaran sponge-grade fossils^43^, modern demosponge larvae^40,41^, colonial protists^39,69^ (Table S2). Most features are shared with red algae, but affinity to other epithelia-bearing groups cannot be excluded.

Considering the emergence of coeval fossils with epithallus-grade organisation^36^, as well as preservation and environmental context, *Cyathinema* likely represents the oldest organically-preserved organism with a layer of differentiated external cells outside of the windows of exceptional preservation, which improves the understanding of biological complexity in the early Ediacaran.

## Methods

Samples D14-N1, D14-N2 and D16-HA-69 were collected from the Nyborg Formation outcrop south of Árasulluokta (N70°32.842′ E028°06.205′), and sample D16-HA-74 from the Guvssájohka valley section (N70°33.451′, E028°04.736′), as a part of Digermulen Early Life Research Group expeditions. Rock samples were collected 5 cm (D16-HA-69), 20 cm (D14-N1), 2 m (D14-N2), and 8 m (D16-HA-74) below the base of the Mortensnes diamictite. All samples were cleaned with 30% hydrochloric acid, then macerated in 40% hydrofluoric acid, following the modified palynological preparation method^97^. The residue was filtered through 10 and 25 μm meshes, rinsed with deionized water, and stored in acetone to prevent the growth of contaminants. Larger fossil fragments were picked from the residue with a micropipette (following the low-manipulation maceration method^98^). Thin sections (30 μm) were cut to check if the recovered organic fossils are syngeneic and not contamination. Microfossils were observed and imaged with Olympus BX50142 light microscope with an Olympus UC30 camera, as well as Zeiss Supra35VP Genesis 4000 and FEI Quanta 400 field emission scanning electron microscopes. Elemental analysis was performed using Genesis 4000 Cryospec EDAX (at 10 kV) attached to Zeiss Supra35VP SEM at Evolutionary Biology Centre, Uppsala University. Nicolet Magna 850 Raman Spectrometer at Materials Research Laboratory, UC Santa Barbara was used to further assess fossils’ composition. Target area on the fossil was identified with a light microscope and excited using a green laser (λ = 532 nm) of low output power (25 mW) to minimize the damage to organic tissue. Rock samples and glass slides with mounted fossils are deposited in the palaeontological collection of the Arctic University Museum of Norway (UiT), prefix TSGf. The International Code of Nomenclature for Algae, Fungi, and Plants is followed in description of a new taxon.

## Supplementary information

Supplementary Information File

## Data Availability

All data pertinent to this study and its reported findings can be found in the article itself or the corresponding Supplementary Information file.

## References

[1] Hoffman, P. F. *et al*. Snowball Earth climate dynamics and Cryogenian geology-geobiology. *Sci*. *Adv*. **3** (2017). 10.1126/sciadv.1600983 PMC5677351 29134193

[2] Och LM, Shields-Zhou GA (2012). The Neoproterozoic oxygenation event: Environmental perturbations and biogeochemical cycling. Earth-Science Rev.

[3] Sahoo SK (2016). Oceanic oxygenation events in the anoxic Ediacaran ocean. Geobiology.

[4] Liu AG, Kenchington CG, Mitchell EG (2015). Remarkable insights into the paleoecology of the Avalonian Ediacaran macrobiota. Gondwana Res..

[5] Bowyer F, Wood RA, Poulton SW (2017). Controls on the evolution of Ediacaran metazoan ecosystems: A redox perspective. Geobiology.

[6] Droser ML, Tarhan LG, Gehling JG (2017). The Rise of Animals in a Changing Environment: Global Ecological Innovation in the Late Ediacaran. Annu. Rev. Earth Planet. Sci..

[7] Muscente AD, Boag TH, Bykova N, Schiffbauer JD (2018). Environmental disturbance, resource availability, and biologic turnover at the dawn of animal life. Earth-Science Rev.

[8] Xiao S, Knoll AH, Yuan X, Pueschel CM (2004). Phosphatized multicellular algae in the Neoproterozoic Doushantuo Formation, China, and the early evolution of florideophyte red algae. Am. J. Bot..

[9] Yuan X, Chen Z, Xiao S, Zhou C, Hua H (2011). An early Ediacaran assemblage of macroscopic and morphologically differentiated eukaryotes. Nature.

[10] Ye Q (2015). The survival of benthic macroscopic phototrophs on a Neoproterozoic snowball Earth. Geology.

[11] Grazhdankin D (2014). Patterns of Evolution of the Ediacaran Soft-Bodied Biota. J. Paleontol..

[12] Schiffbauer JD (2016). The Latest Ediacaran Wormworld Fauna: Setting the Ecological Stage for the Cambrian Explosion. GSA Today.

[13] Wood, R. & Penny, A. Substrate growth dynamics and biomineralization of an ediacaran encrusting poriferan. *Proc. R. Soc. B Biol. Sci*. **285** (2018). 10.1098/rspb.2017.1938 PMC5784191 29321296

[14] Knoll AH (2011). The Multiple Origins of Complex Multicellularity. Annu. Rev. Earth Planet. Sci..

[15] Sebé-Pedrós A, Degnan BM, Ruiz-Trillo I (2017). The origin of Metazoa: A unicellular perspective. Nat. Rev. Genet..

[16] Butterfield NJ (2009). Modes of pre-Ediacaran multicellularity. Precambrian Res..

[17] Xiao S, Zhou C, Liu P, Wang D, Yuan X (2014). Phosphatized Acanthomorphic Acritarchs and Related Microfossils from the Ediacaran Doushantuo Formation at Weng’an (South China) and their Implications for Biostratigraphic Correlation. J. Paleontol..

[18] Anderson RP, Macdonald FA, Jones DS, McMahon S, Briggs DEG (2017). Doushantuo-type microfossils from latest Ediacaran phosphorites of northern Mongolia. Geology.

[19] Cunningham JA, Vargas K, Yin Z, Bengtson S, Donoghue PCJ (2017). The Weng’an Biota (Doushantuo Formation): an Ediacaran window on soft-bodied and multicellular microorganisms. J. Geol. Soc. London.

[20] Butterfield NJ, Knoll AH, Swett K (1994). Paleobiology of the Neoproterozoic Svanbergfjellet Formation, Spitsbergen. Foss. Strat.

[21] Orr, P. J. Late Proterozoic-early Phanerozoic ‘taphonomic windows’: The environmental and temporal distribution of recurrent modes of exceptional preservation. In *Reading and Writing of the Fossil Record: Preservational Pathways to Exceptional Fossilization*. *The Paleontological Society Papers*, **20** (eds Laflamme, M., Schiffbauer, J. & Darroch, S. A. F.), 289–313 (2014).

[22] Xiao S (2016). Towards an Ediacaran Time Scale: Problems, Protocols, and Prospects. Episodes.

[23] Pu JP (2016). Dodging snowballs: Geochronology of the Gaskiers glaciation and the first appearance of the Ediacaran biota. Geology.

[24] Anderson RP, McMahon S, Bold U, Macdonald FA, Briggs DEG (2017). Palaeobiology of the early Ediacaran Shuurgat Formation, Zavkhan Terrane, south-western Mongolia. J. Syst. Palaeontol.

[25] Zang W, Walter MR (1989). Latest Proterozoic plankton from the Amadeus Basin in Central Australia. Nature.

[26] Grey K (2005). Ediacaran palynology of Australia. Assoc. Australas. Palaeontol.

[27] Agić, H. *et al*. Life through the ‘Varanger Ice Ages’: Microfossil record of late Neoproterozoic glacial-interglacial units from Arctic Norway. *Geol*. *Soc*. *Am*. *Abstr*. *with Programs* **50** (2018).

[28] Rice, A. H. N., Edwards, M. B., Hansen, T. A., Arnaud, E. & Halverson, G. P. Glacigenic rocks of the Smalfjord and Mortensnes Formations, Vestertana Group, E. Finnmark, Norway. In *The Geological Record of* Neoproterozoic *Glaciations* (eds Arnaud, E., Halverson, G. & Shields-Zhou, G.) 593–602 (Geological Society of London, 2011).

[29] Halverson GP, Hoffman PF, Schrag DP, Maloof AC, Rice AHN (2005). Toward a Neoproterozoic composite carbon-isotope record. Bull. Geol. Soc. Am.

[30] Banks N, Edwards MB, Geddes WP, Hobday DK, Reading HG (1971). Late Precambrian and Cambro-Ordovician sedimentation in east Finnmark. Norges Geol. Undersøkelse.

[31] Farmer (1992). Ediacaran fossils from the Innerelv Member (late Proterozoic) of the Tanafjorden area, northeastern Finnmark. Geol. Mag..

[32] Högström AES, Jensen S, Palacios T, Ebbestad JOR (2013). New information on the Ediacaran-Cambrian transition in the Vestertana Group, Finnmark, northern Norway, from trace fossils and organic-walled microfossils. Nor. Geol. Tidsskr.

[33] Jensen, S. *et al*. New occurrences of *Palaeopascichnus* from the Stáhpogieddi Formation, Arctic Norway, and their bearing on the age of the Varanger Ice Age. **10**, 1–10 (2018).

[34] Vidal G (1981). Micropalaeontology and biostratigraphy of the Upper Proterozoic and Lower Cambrian sequence in East Finnmark, Northern Norway. Norges Geol. Undersøkelse.

[35] Lahfid A (2010). Evolution of the Raman spectrum of carbonaceous material in low-grade metasediments of the Glarus Alps (Switzerland). Terra Nov.

[36] Butterfield NJ (1990). Organic Preservation of Non-Mineralizing Organisms and the Taphonomy of the Burgess Shale. Paleobiology.

[37] Xiao S, Zhang Y, Knoll AH (1998). Three-dimensional preservation of algae and animal embryos in a Neoproterozoic phosphorite. Nature.

[38] Galicia-García C, Robinson NM, Okolodkov YB (2013). New records of red algae (Rhodophyta) for Cabezo Reef, National Park Sistema Arrecifal Veracruzano, Gulf of Mexico. Acta Bot. Mex..

[39] Jeuck A, Arndt H (2013). A Short Guide to Common Heterotrophic Flagellates of Freshwater Habitats Based on the Morphology of Living Organisms. Protist.

[40] Ereskovsky, A. V. *The Comparative Embryology of Sponges*. (Springer Science+Business Media, 2010).

[41] Lavrov AI, Kosevich IA (2018). Stolonial Movement: A New Type of Whole-Organism Behavior in Porifera. Biol. Bull..

[42] Mah JL, Leys SP (2017). Think like a sponge: The genetic signal of sensory cells in sponges. Dev. Biol..

[43] Yin Z (2015). Sponge grade body fossil with cellular resolution dating 60 Myr before the Cambrian. Proc. Natl. Acad. Sci..

[44] Faegri K (1963). Organ and Form Genera: Significance and Nomenclatural Treatment. Taxon.

[45] Muscente AD, Hawkins AD, Xiao S (2015). Fossil preservation through phosphatization and silicification in the Ediacaran Doushantuo Formation (South China): A comparative synthesis. Palaeogeogr. Palaeoclimatol. Palaeoecol.

[46] Russell G, Veltkamp CJ (1984). Epiphyte survival on skin-shedding macrophytes. Mar. Ecol. Ser.

[47] Fletcher RL, Callow ME (1992). The settlement, attachment and establishment of marine algal spores. Br. Phycol. J.

[48] Johnson CR, Mann KH (1986). The crustose coralline alga, *Phymatolithon* Foslie, inhibits the overgrowth of seaweeds without relying on herbivores. J. Exp. Mar. Bio. Ecol.

[49] Pueschel CM, Miller TJ (1996). Reconsidering prey specializations in an algal-limpet grazing mutualism: epithallial cell development in *Clathromorphum circumscriptum* (Rhodophyta, Corallinales). J. Phyc.

[50] Gargiulo GM, De Masi F, Tripodi G (1987). Structure and reproduction of *Gracilaria longa* sp. nov. (Rhodophyta, Gigartinales) from the Mediterranean Sea. G. Bot. Ital.

[51] Mariath R, Riosmena-Rodriguez R, Figueiredo M (2012). *Lithothamnion steneckii* sp. nov. and *Pneophyllum conicum*: new coralline red algae (Corallinales, Rhodophyta) for coral reefs of Brazil. Algae.

[52] Maldonado M (2004). Choanoflagellates, choanocytes, and animal multicellularity. Invertebr. Biol.

[53] Dayel MJ, King N (2014). Prey capture and phagocytosis in the choanoflagellate *Salpingoeca rosetta*. PLoS One.

[54] Xiao S, Zhou C, Liu P, Wang D, Yuan X (2014). Phosphatized acanthomorphic acritarchs and related microfossils from the Ediacaran Doushantuo Formation at Weng’an (South China) and their implications for biostratigraphic correlation. J. Paleontol..

[55] Zhu S (2016). Decimetre-scale multicellular eukaryotes from the 1.56-billion-year-old Gaoyuzhuang Formation in North China. Nat. Commun..

[56] Honegger, R., Edwards, D., Axe, L. & Strullu-Derrien, C. Fertile *Prototaxites taiti*: A basal ascomycete with inoperculate, polysporous asci lacking croziers. *Philos*. *Trans*. *R*. *Soc*. *B Biol*. *Sci*. **373** (2018). 10.1098/rstb.2017.0146 PMC5745340 29254969

[57] Taylor, T. N., Krings, M. & Taylor, E. L. *Fossil fungi*. (Academic Press, 2014).

[58] Yuan X, Xiao S, Taylor T (2005). Lichen-like symbiosis 600 million Years Ago. Science (80-.).

[59] Smith MR (2016). Cord-forming Palaeozoic fungi in terrestrial assemblages. Bot. J. Linn. Soc..

[60] Berbee ML, James TY, Strullu-Derrien C (2017). Early Diverging Fungi: Diversity and Impact at the Dawn of Terrestrial Life. Annu. Rev. Microbiol..

[61] Loron CC (2019). Early fungi from the Proterozoic era in Arctic Canada. Nature.

[62] Erwin DH (2011). The Cambrian conundrum: Early divergence and later ecological success in the early history of animals. Science.

[63] Filipiak P, Szaniawski H (2016). Nematophytes from the Lower Devonian of Podolia, Ukraine. Rev. Palaeobot. Palynol..

[64] Smith MR, Butterfield NJ (2013). A new view on *Nematothallus*: Coralline red algae from the Silurian of Gotland. Palaeontology.

[65] Edwards D, Axe L, Honegger R (2013). Contributions to the diversity in cryptogamic covers in the mid-Palaeozoic: *Nematothallus* revisited. Bot. J. Linn. Soc..

[66] Dos Reis M (2015). Uncertainty in the Timing of Origin of Animals and the Limits of Precision in Molecular Timescales. Curr. Biol..

[67] King N, Rokas A (2017). Embracing Uncertainty in Reconstructing Early Animal Evolution. Curr. Biol..

[68] Leadbeater BSC (2008). Choanoflagellate evolution: the morphological perspective. Protistology.

[69] Martin, G. W. & Alexopoulos, C. J. *The Myxomycetes*. (University of Iowa Press, 1969).

[70] Dörfelt H, Schmidt AR, Ullmann P, Wunderlich J (2003). The oldest fossil myxogastroid slime mould. Mycol. Res..

[71] Hughes AJ, Gooday AJ (2004). Associations between living benthic foraminifera and dead tests of *Syringammina fragilissima* (Xenophyophorea) in the Darwin Mounds region (NE. *Atlantic)*. Deep. Res.

[72] Kolesnikov AV (2018). The oldest skeletal macroscopic organism *Palaeopascichnus linearis*. Precambrian Res..

[73] Botting JP, Muir LA (2017). Early sponge evolution: A review and phylogenetic framework. Palaeoworld.

[74] Sperling EA, Robinson JM, Pisani D, Peterson KJ (2010). Sperling *et al* 2010 Where’s the glass- Biomarkers, molecular clocks, and microRNAs suggest a 200 - Myr missing Precambrian fossil record of siliceous sponge spicules.pdf. Geobiology.

[75] Brocks JJ (2016). Early sponges and toxic protists: possible sources of cryostane, an age diagnostic biomarker antedating Sturtian Snowball Earth. Geobiology.

[76] Zumberge J. Alex, Love Gordon D., Cárdenas Paco, Sperling Erik A., Gunasekera Sunithi, Rohrssen Megan, Grosjean Emmanuelle, Grotzinger John P., Summons Roger E. (2018). Demosponge steroid biomarker 26-methylstigmastane provides evidence for Neoproterozoic animals. Nature Ecology & Evolution.

[77] Antcliffe JB, Callow RHT, Brasier MD (2014). Giving the early fossil record of sponges a squeeze. Biol. Rev..

[78] Muscente AD, Marc Michel F, Dale JG, Xiao S (2015). Assessing the veracity of Precambrian ‘sponge’ fossils using *in situ* nanoscale analytical techniques. Precambrian Res..

[79] Nettersheim BJ (2019). Putative sponge biomarkers in unicellular Rhizaria question an early rise of animals. Nat. Ecol. Evol.

[80] Ehrlich H (2013). Discovery of 505-million-year old chitin in the basal demosponge *Vauxia gracilenta*. Sci. Rep.

[81] Smith CL, Reese TS (2016). Adherens junctions modulate diffusion between epithelial cells in *Trichoplax adhaerens*. Biol. Bull..

[82] Zhang Y (1998). Interpreting Late Precambrian Microfossils. Science.

[83] Liu P (2014). Ediacaran acanthomorphic acritarchs and other microfossils from chert nodules of the upper Doushantuo Formation in the Yangtze Gorges area, South China. J. Paleontol..

[84] Yang, E. *et al*. Divergence time estimates and evolution of major lineages in the florideophyte red algae. *Sci*. *Rep*. **6**, 21361. 10.1038/srep21361 PMC4759575 26892537

[85] Martindale RC, Corsetti FA, Bottjer DJ, Senowbari-Daryan B (2012). Microbialite Fabrics and Diminutive Skeletal Bioconstructors in Lower Norian Summit Point Reefs, Oregon, United States. Palaios.

[86] Hind KR, Gabrielson PW, P. Jensen C, Martone PT (2016). *Crusticorallina* gen. nov., a nongeniculate genus in the subfamily Corallinoideae (Corallinales, Rhodophyta). J. Phycol..

[87] Dawes CJ, Scott FM, Bowler E (1961). A Light- and Electron-Microscopic Survey of Algal Cell Walls. I. Phaeophyta and Rhodophyta. Am. J. Bot..

[88] Nash, M. C. & Adey, W. Multiple phases of Mg-calcite in crustose coralline algae suggest caution for temperature proxy and ocean acidification assessment: lessons from the ultrastructure and biomineralization in *Phymatolithon* (Rhodophyta, Corallinales). *J*. *Phycol*. **53** (2017). 10.1111/jpy.12559 28671731

[89] Delivopoulos S (2011). Ultrastructure of trichoblasts in the red alga *Osmundea spectabilis* var. *spectabilis* (Rhodomelaceae, Ceramiales). Eur. J. Phycol..

[90] Butterfield NJ (2000). *Bangiomorpha pubescens* n. gen., n. sp.: implications for the evolution of sex, multicellularity, and the Mesoproterozoic/Neoproterozoic radiation of eukaryotes. Paleobiology.

[91] Gibson TM (2018). Precise age of *Bangiomorpha pubescens* dates the origin of eukaryotic photosynthesis. Geology.

[92] Bengtson, S., Sallstedt, T., Belivanova, V. & Whitehouse, M. Three-dimensional preservation of cellular and subcellular structures suggests 1.6 billion-yearold crown-group red algae. *PLoS Biology* **15** (2017). 10.1371/journal.pbio.2000735 PMC5349422 28291791

[93] Ray JS (2006). Age of the Vindhyan Supergroup: A review of recent findings. J. Earth Syst. Sci..

[94] Cohen PA, Macdonald FA, Pruss S, Matys E, Bosak T (2015). Fossils of Putative Marine Algae From the Cryogenian Glacial Interlude of Mongolia. Palaios.

[95] Tyler S (2003). Epithelium–The Primary Building Block for Metazoan Complexity. Integr. Comp. Biol..

[96] Belahbib H (2018). New genomic data and analyses challenge the traditional vision of animal epithelium evolution. BMC Genomics.

[97] Grey, K. A modified palynological preparation technique for the extraction of large Neoproterozoic acanthomorph acritarchs and other acid insoluble microfossils. *Geol*. *Surv*. *West*. *Aust*. 1–23 (1999).

[98] Butterfield NJ, Harvey THP (2012). Small carbonaceous fossils (SCFs): A new measure of early paleozoic paleobiology. Geology.

