# Supplementary material for: Organically-preserved multicellular eukaryote from the early Ediacaran Nyborg Formation, Arctic Norway
Source: Sci Rep. 2019 Oct 10;9:14659. doi: 10.1038/s41598-019-50650-x (PMC6787099; doi:10.1038/s41598-019-50650-x)
Supplement: Supplementary file 1 — Supplementary Information File [file 41598_2019_50650_MOESM1_ESM.pdf]

Organically-preserved multicellular eukaryote from the early Ediacaran Nyborg Formation, Arctic Norway

Heda Agić[1,2], Anette E. S. Högström[3], Małgorzata Moczydłowska[2], Sören Jensen[4], Teodoro Palacios[4], Guido Meinhold[5,6], Jan Ove R. Ebbestad[7], Wendy L. Taylor[8] & Magne Høyberget[9]

1 Department of Earth Science, University of California Santa Barbara, CA 93106, USA.

2 Department of Earth Sciences, Uppsala University, 752 36 Uppsala, Sweden.

3 Tromsø University Museum, UiT - The Arctic University of Norway, N-9073 Tromsø, Norway.

4 Área de Paleontología, Universidad de Extremadura, E-06006 Badajoz, Spain.

5 School of Geography, Geology and the Environment, Keele University, Keele, ST5 5BG, UK.

6 Department of Sedimentology and Environmental Geology, University of Göttingen, Goldschmidtstraße 3, 37077, Göttingen, Germany.

7 Museum of Evolution, Uppsala University, 752 36 Uppsala, Sweden.

8 Department of Geological Sciences, University of Cape Town, Rondebosch 7701, South Africa.

9 Rennesveien 14, N-4513 Mandal, Norway.

Correspondence should be addressed to H.A. (e-mail: hagic@geol.ucsb.edu)

Supplementary Information

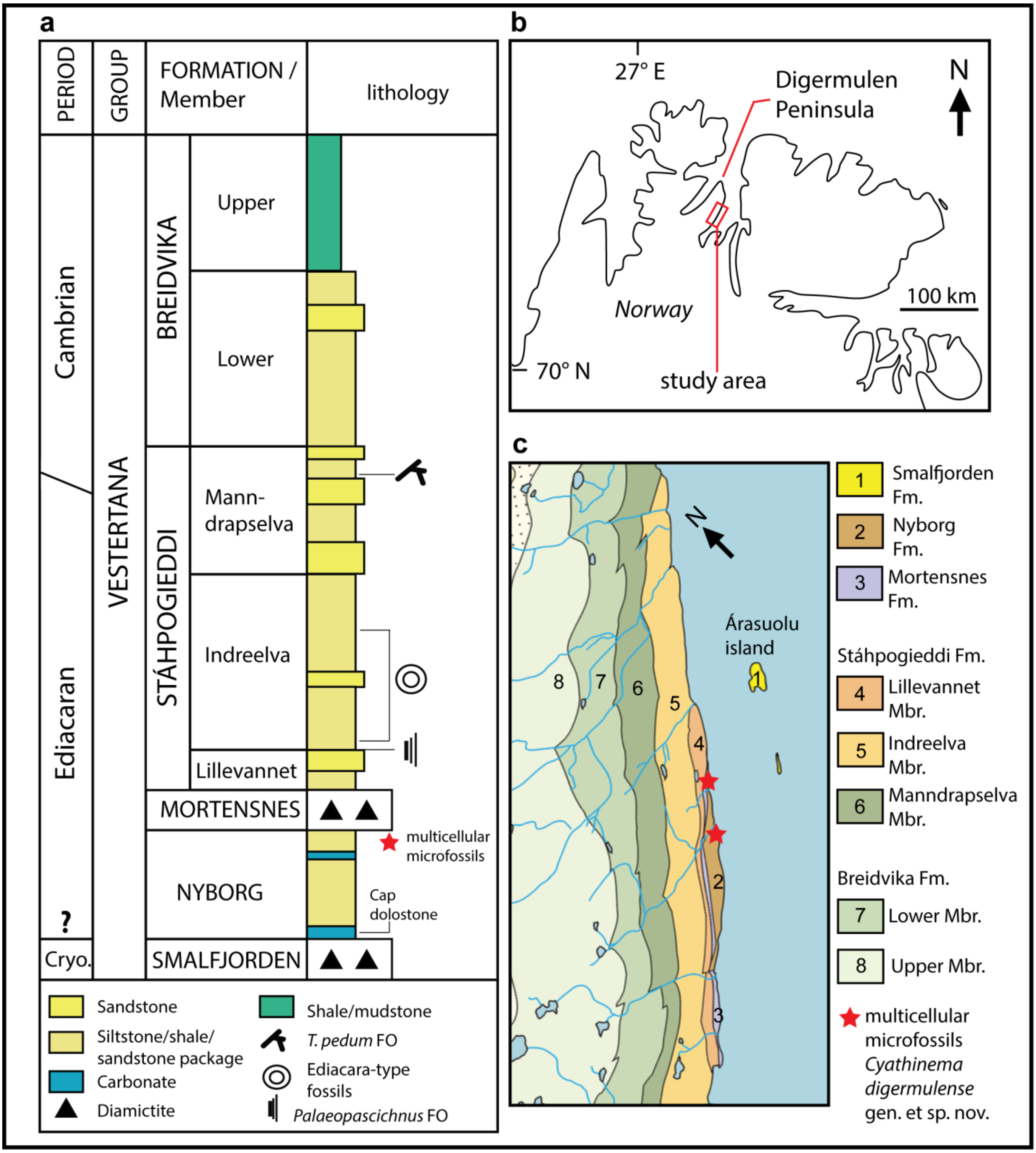

Supplementary Figure 1. Location and stratigraphy of the Cryogenian–Ediacaran Vestertana Group in Arctic Norway. Cyathinema digermulense *gen. et sp. nov. was recovered from siliciclastic rocks of the uppermost Nyborg Formation.* ***(a)*** *Schematic stratigraphy of the Vestertana Group. The Smalfjorden diamictite has been correlated with the Marinoan glaciation.* ***(b)*** *Geographic position of the Digermulen Peninsula.* ***(c)*** *Geological map of the southeastern part of the Digermulen Peninsula and location of the Nyborg Formation sediments.*

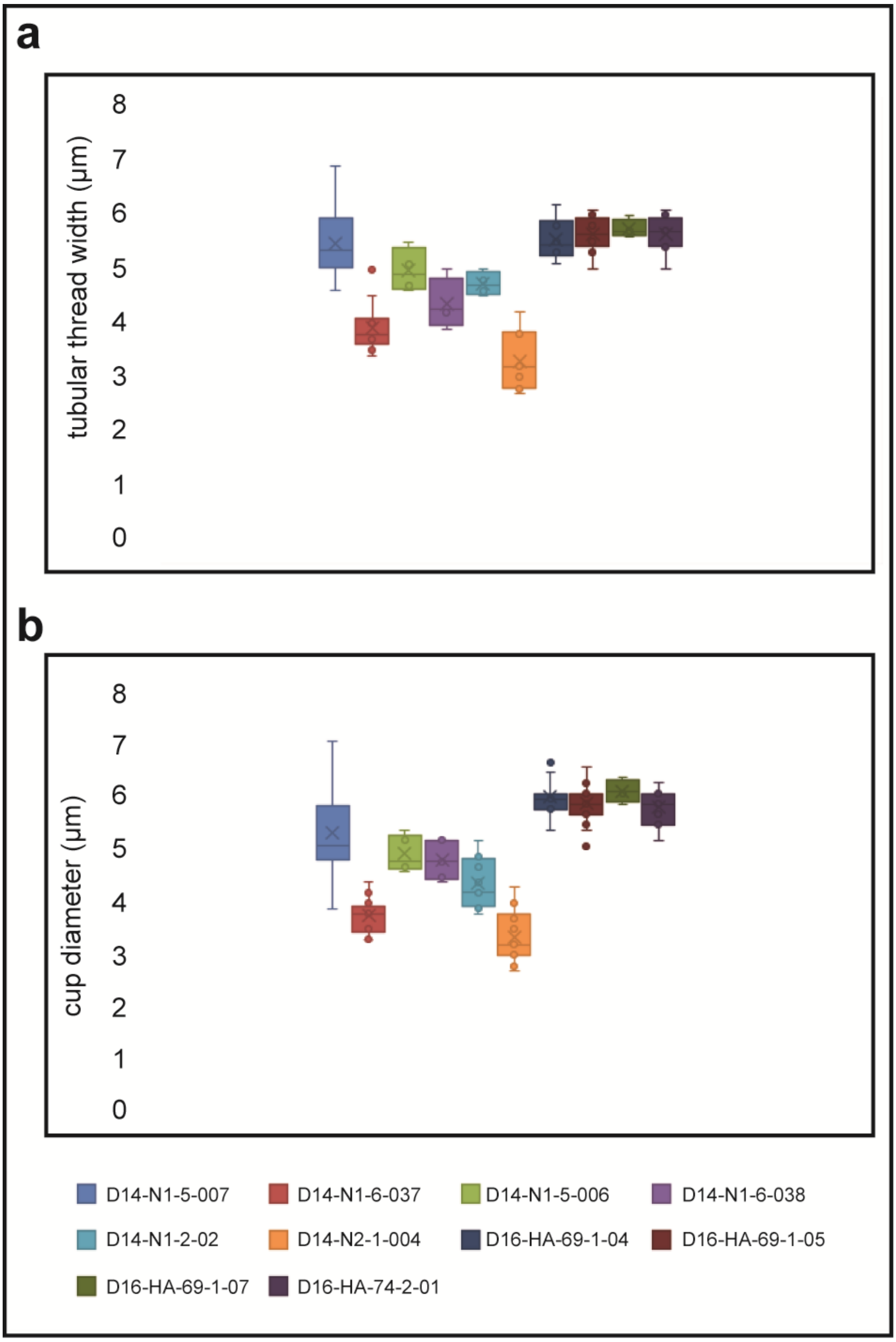

Supplementary Figure 2. Variation in size of individual morphological elements between specimens of* Cyathinema digermulense *gen. et sp. nov*.** *The box-and-whiskers plots show the range of sizes of characteristics (tubular threads and cup-shaped structures) in a statistical population of* Cyathinema *specimens. Plots for individual specimens are colour-coded according to the legend given at the bottom of the figure.* ***(a) *Variation in the width of tubular threads between individual specimens. Number of measured tubular threads = 91.* ***(b)*** *Variation in diameter of cup-shaped termini per*

individual specimen. Number of measured cup-shaped structures = 196. Both the cup and tube structures are usually uniform in size on a given specimen, and the variation may be attributed to poor preservation and/or deflation upon being mounted on glass slides.

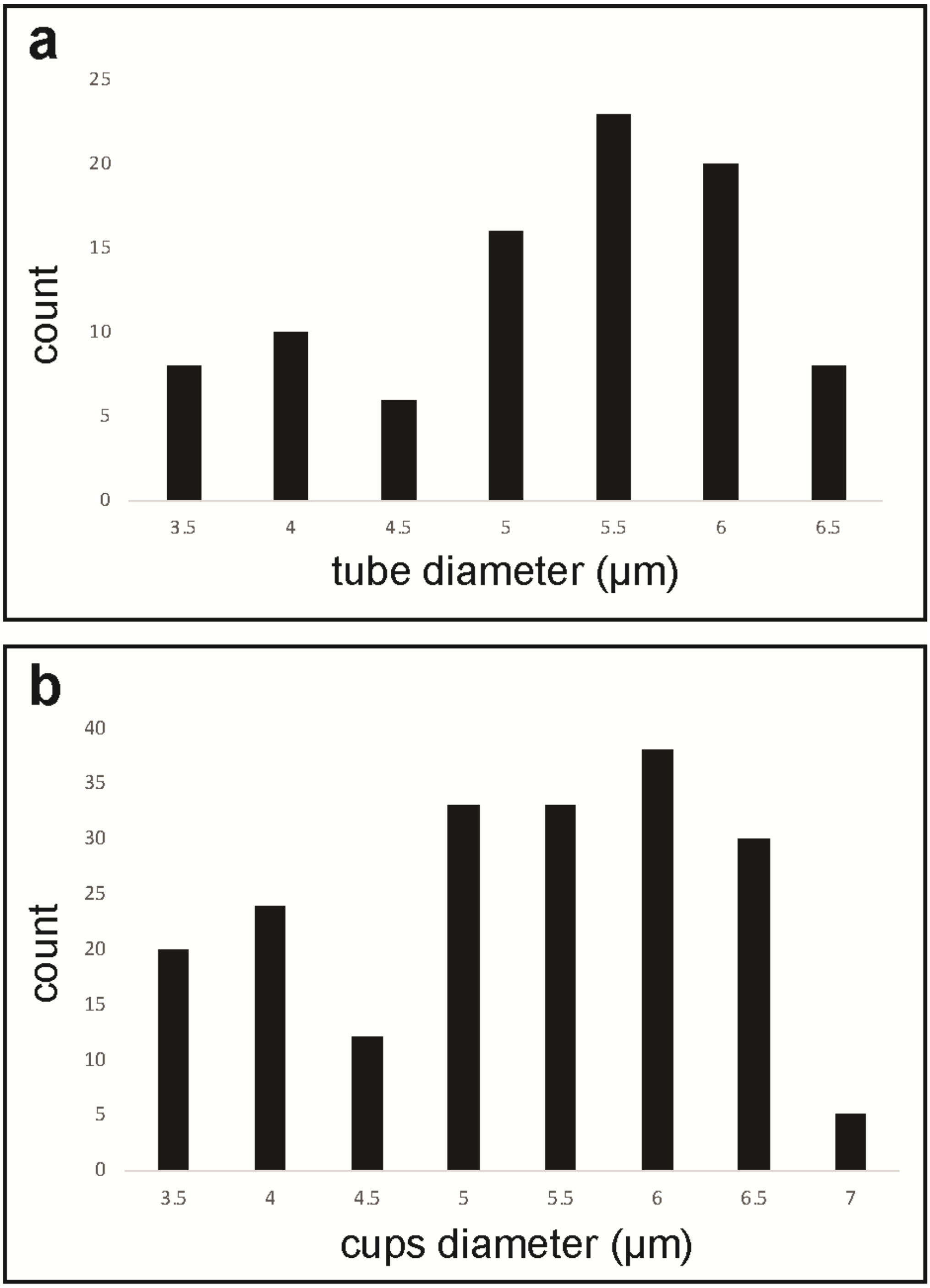

Supplementary Figure 3. Measured dimensions of the elements of* Cyathinema digermulense *gen. et sp. nov., tubes and their cup-shaped openings*. (a)** *Histogram of the width of the tubular threads (n = 93).* ***(b) *Histogram of the diameter of cup-shaped structures (n = 197).*

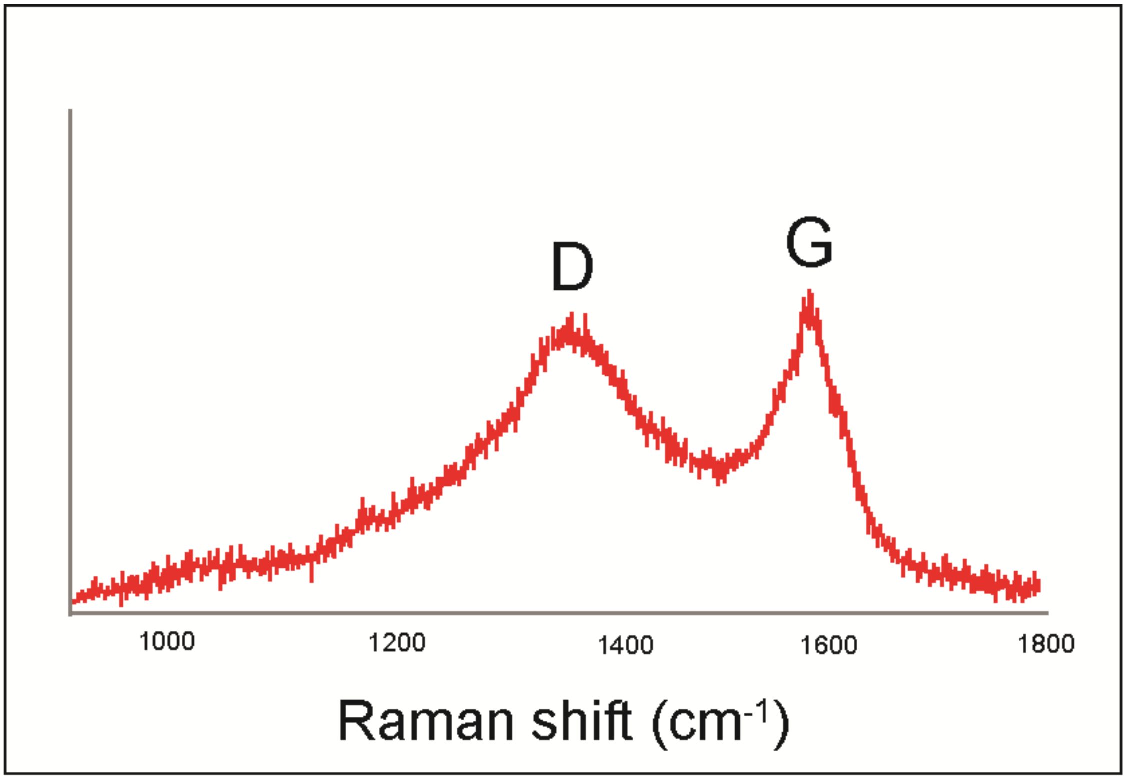

Supplementary Figure 4. Raman spectrum of* Cyathinema digermulense *gen. et sp. nov. *The analysis was performed on specimen TG1f-D16-HA-74-2-01. Intensity of the spectrum suggests fluorescence of the analysed material including the background signal, yet diagnostic peaks are still recognizable. Prominent peaks at 1367 cm^{-1} and 1621 cm^{-1} correspond to the disordered (D) and graphitic (G) bands which are indicative of organic matter and show that the fossil originally had carbonaceous constituents. An intense D-band follows increasing maturation, which shows that the fossil organic matter underwent thermal alteration under low epizonal metamorphic conditions (per[1,2]). The specimen was destroyed by the analysis.*

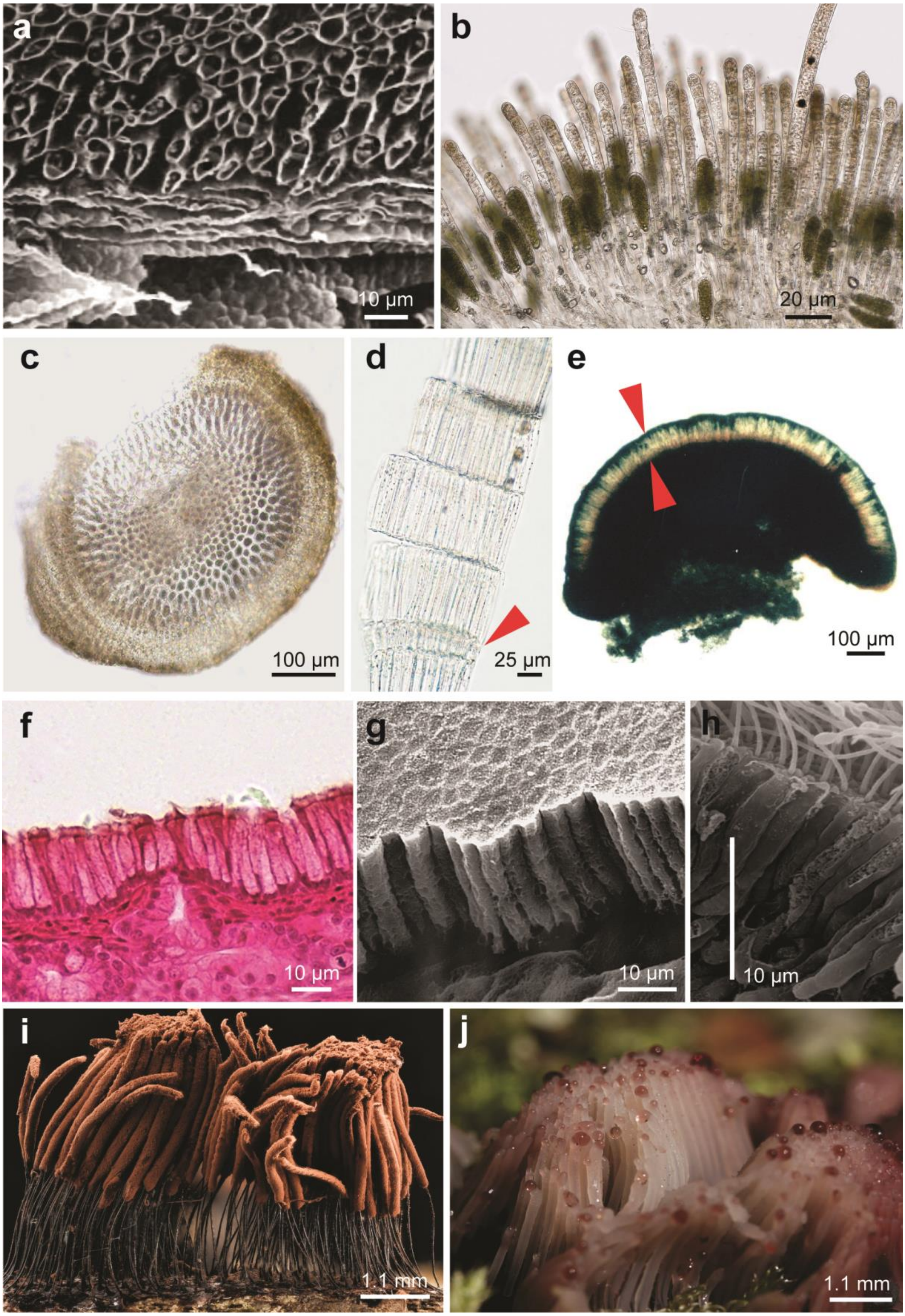

Supplementary Figure 5. Organisms and organismal structures similar to **Cyathinema digermulense** ***gen. et sp. nov. (a)*** *Scanning electron micrograph of an epithallus of* Himanthalia elongata*. Epithallus consists of tubular cells with open terminations, corresponding in size to*

Cyathinema *tubes. Modified with permission from ref*[3], *©Inter-Research 1984.* ***(b)*** *Transmitted light photomicrograph of outer cell layer sporangium of the brown alga* Elachista scutulata *(Phaeophyceae) from the shore of Outer Hebrides, UK. Sporangium tubes are closed distally, but open during zoospore release. Image courtesy of: Chris Johnson.* ***(c-d)*** *Coralline alga* Amphiroa fragilissima *(Florideophyceae) from Cabezo reef, Gulf of Mexico, under transmitted light microscope. Images courtesy of: Yuri Okolodkov.* ***(c)*** *Transverse section of* Amphiroa *thallus showing cortical epithallial cells.* ***(d)*** *Longitudinal section of the epithallus, consisting of rows of stacked longer and shorter (arrow) tubular cells, corresponding in size to* Cyathinema *tubes.* ***(e)*** *Epidermal tissue of a terrestrial fungus* Bacidia schweinitzii *(Ascomycota) from Maine, USA. Arrows indicate an outer layer of differentiated tube-like outer cells which consists of the dark-green epihymenium and a reddish-brown hypothecium. Credit: Ed Uebel. Image used from Wikimedia Commons archive and shared under Creative Commons 4.0. Note that* Bacidia *is a terrestrial organism growing on tree barks and is unlikely to be related to marine* Cyathinema, *but an outer layer of stacked tubular cells is common in multicellular eukaryotes.* ***(f)*** *An example of pseudostratified columnar epithelium in Metazoa. Cross section of mammalian trachea. Magnification 200x. Credit: Berkshire Community College Bioscience Image Library, http://blogs.berkshirecc.edu/bccoer/animals/#Epithelial_Tissues, image modified and shared under Creative Commons 4.0.* ***(g)*** *Scanning electron micrograph of a simple columnar epithelium. Image courtesy of: Roger Wagner. Simple columnar epithelia are a single layer of epithelial cells present in a variety of animal tissues.* ***(h)*** *Tubular epithelial cells with cilia in parenchymella larva of* Scopalina lophyropoda *(Demospongiae). Reused with permission from ref*[4] *©Canadian Science Publishing or its licensors, and courtesy of Manuel Malonado.* ***(i-j)*** *Stacked tubes of sporangia of slime mold* Stemonitis *sp. (Mycetozoa), from the Adirondack Mountains, USA* ***(i)***, *and Hilden, Germany* ***(j)***. *Credit: Dejen Mengis. Images taken from Wikimedia Commons and shared under Creative Commons 4.0.* Stemonitis *becomes multicellular during a part of its life cycle (reproduction), but it is a terrestrial organism and an order of magnitude larger than* Cyathinema. *New fossils from the Nyborg Formation share similarities with various groups of multicellular eukaryotes, more specifically with organisms that possess an outer layer of differentiated, tubular cells.*

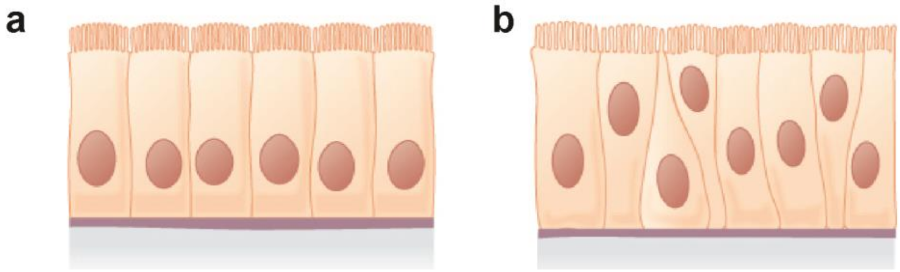

Supplementary Figure 6. Schematic illustrations of simple types of differentiated cell layers (epithelial cells). ***(a)*** *Simple columnar epithelium (Eumetazoa). This type of epithelial cells is common in animals, and in mammals it usually lines organs of the digestive tract.* ***(b)*** *Pseudostratified columnar epithelium present in animal respiratory, intestinal, and reproductive organs. Illustrations are adapted from* Anatomy & Physiology. *Credit: OpenStax College, https://cnx.org/contents/FPtK1zmh@6.27:oWqVExrJ@3/Epithelial-Tissue, shared under Creative Commons 4.0.*

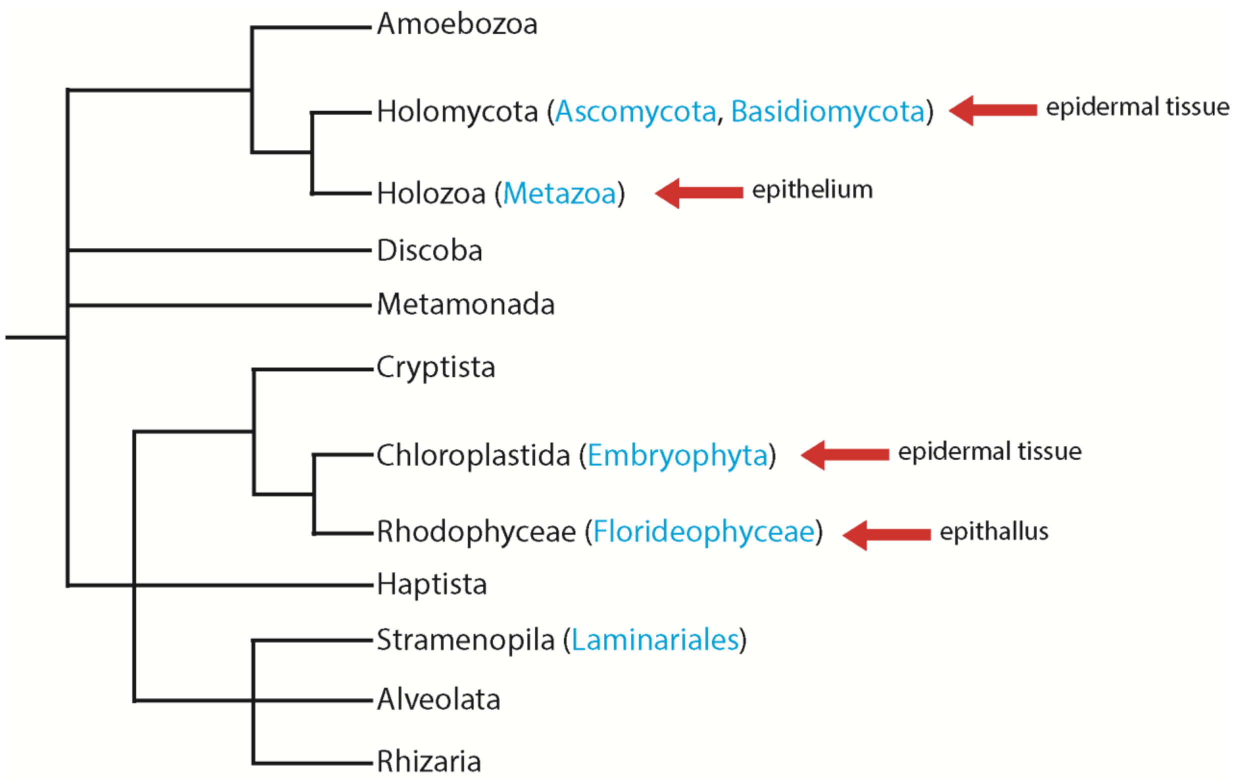

Supplementary Figure 7. Distribution of multicellularity and differentiated external cell layers (epithelia/epithalli) among eukaryotes*. Eukaryotic clades that have independently evolved multicellularity are marked in blue. Red arrows indicate clades that possess a layer of distinct outer cells: epithallus/epithelium/epidermis. Phylogenetic tree of eukaryotes is modified and redrawn from refs*[5,6]*. Multicellularity is present in two groups of fungi (Holomycota): sac fungi (ascomycetes) and basidiomycetes. Both possess an outer layer of differentiated, tube-like cells – epidermis. Epithelium is an outer layer of a body surface present in Eumetazoa (Holozoa), and is one of the four tissue types in animals. Epithelia vary in size and types, but simple columnar epithelium is present in most animals. Florideophyte red algae contain a differentiated outer cell layer called epithallus which is used in avoidance of overgrowth or sedimentary disturbance, and limitation of fouling. Land plants (embryophyta) possess a complex layer of epidermal cells covering roots, stems, leaves and flowers, which are used in nutrient uptake, gas exchange, and water-loss mitigation. Differentiated outer cell layer among the brown algae (Laminariales) occurs as sporangia. Like multicellularity, a differentiated outer layer of epithelial cells likely arose independently among various groups of multicellular eukaryotes.* Cyathinema digermulense *gen. et sp. nov. from the early Ediacaran Nyborg Formation consists of stacked tubes, an arrangement that is shared by organisms with a layer of differentiated outer cells (epithelium/epithallus/epidermis). Regardless of the biological affinity of the Nyborg fossils, along with the Ediacaran red algae, they were likely one of the earliest organisms with an outer cell layer (epithelium* sensu lato*).*

a

Macerate or thin section	Specimen #	Mean tube width (µm)	Mean tube length (µm)	Mean cup diametre (µm)	Overall meshwork size (length; µm)
**macerate**	TSGf18420c	5.47	17.2	5.37	113
**macerate**	TSFf18423f	3.92	12.53	3.78	98
**macerate**	TSGf18420a				147
**macerate**	TSGf18420b	4.97	7.7	4.94	68
**macerate**	D14-N1-2-001	5.2			202
**macerate**	TSGf18423g	4.35	8.5	4.82	52
**macerate**	D14-N1-6-025				224
**macerate**	TSGf18424	3.29	10.12	3.34	84
**macerate**	D14-N1-2-02	4.72	8.7	4.4	137
**macerate**	TSf18422	6.2			210
**macerate**	TSGf18425a	5.55	19.7	6.03	327
**macerate**	TSGf18425b	5.64	20.25	5.9	212
**macerate**	TSGf18425c	5.75	17.75	6.15	231
**macerate**	D16-HA-74-2-01	5.65	20	5.83	107
**Mean size of macerated specimens**		**5.06**	**14.24**	**5.06**	**158**
**St. deviation of macerated specimens**		**0.85**	**5.23**	**0.98**	**78.15**
**thin section**	TSGf18424a				74.5
**thin section**	TSGf18424b				68
**thin section**	TSGf18424c				152
**thin section**	TSGf18424d				413
**thin section**	TSGf18426a				298
**thin section**	TSGf18426b				284.5
**thin section**	TSGf18427a				167
**thin section**	TSGf18427b				297
**thin section**	D14-N1-TS3-01				379
**thin section**	D14-N1-TS3-02				184
**Mean size of specimens in thin sections**		231.7			
**Mean fossil size (all specimens)**		192.26			
**Standard deviation (all specimens)**		120.34			

b

***Cyathinema digermulense* specimen #**	**Tube width (µm)**	**Tube length (µm)**	**Cup diameter (µm)**	**Overall specimen size (µm)**	**Inner opening diameter (µm)**
**TSGf18420c**	5.1		5.2	133.3	2

**TSGf18420c**	5	14	5.1		1.8
**TSGf18420c**	6.1		6		2
**TSGf18420c**	4.8		5.8		
**TSGf18420c**	5.1	13.5	5.2		
**TSGf18420c**	4.6		6.1		1.1
**TSGf18420c**	5.7		5.2		
**TSGf18420c**	5.1		5.1		
**TSGf18420c**	6.5	17.2	5.2		1.8
**TSGf18420c**	4.9		4.8		0.9
**TSGf18420c**	6		5.4		
**TSGf18420c**	4.8		5.4		
**TSGf18420c**	5.7		6.8		
**TSGf18420c**	5.8	22.5	5.1		1.8
**TSGf18420c**	6.4		7.1		2.2
**TSGf18420c**	6.1		6		
**TSGf18420c**	5.5	23.3	5		1.2
**TSGf18420c**	5.1		5.1		
**TSGf18420c**	6.9		5		2
**TSGf18420c**	5.3		4.7		
**TSGf18420c**	5.8	13	5.4		
**TSGf18420c**	5.2	14.5	5.2		1.6
**TSGf18420c**	5.5		5		
**TSGf18420c**	4.9		6.2		
**TSGf18420c**	4.8	12.8	4.8		
**TSGf18420c**	6		4		
**TSGf18420c**	5.4		4.8		
**TSGf18420c**	5.3	24	5		
**TSGf18420c**			4.8		
**TSGf18420c**			3.9		
**TSGf18420c**			4.7		
**TSGf18420c**			4.8		
**TSGf18420c**			4.8		
**TSGf18420c**			4.6		
**TSGf18420c**			5		
**TSGf18420c**			4.9		
**TSGf18420c**			5.1		
**TSGf18420c**			5.2		
**TSGf18420c**			4.9		0.9
**TSGf18420c**			5.8		
**TSGf18420c**			4.9		
**TSGf18420c**			6.2		
**TSGf18420c**			6		1.9
**TSGf18420c**			5.7		
**TSGf18420c**			5.6		
**TSGf18420c**			5.6		
**TSGf18420c**			5.1		
**TSGf18420c**			5.6		
**TSGf18420c**			5.2		
**TSGf18420c**			5.1		
**TSGf18420c**			4.9		0.8

**TSGf18420c**			5.1		1.2
**TSGf18420c**			4.8		1
**TSGf18420c**			5		1.3
**TSGf18420c**			5.2		1.3
**TSGf18420c**			4.4		0.8
**TSGf18420c**			4.8		0.9
**TSGf18420c**			4.6		0.8
**TSGf18420c**			4.4		0.7
**TSGf18420c**			4.4		0.7
**TSGf18420c**			6.7		1.9
**TSGf18420c**			7		2
**TSGf18420c**			5.8		1.8
**TSGf18420c**			5.8		
**TSGf18420c**			6.2		
**TSGf18420c**			5.9		
**TSGf18420c**			6.5		
**TSGf18420c**			5.7		
**TSGf18420c**			6.2		
**TSGf18420c**			6.3		
**TSGf18420c**			6.2		
**TSGf18420c**			6.2		
**TSGf18420c**			5		
**TSGf18420c**			5.7		
**TSGf18420c**			5.8		
**TSGf18420c**			6.1		
**TSGf18420c**			6.3		
**TSGf18423f**	3.5	12	3.6	98	0.7
**TSGf18423f**	5		3.9		0.7
**TSGf18423f**	3.7	12.7	3.9		0.9
**TSGf18423f**	3.8		3.8		0.6
**TSGf18423f**	4.1		4		1
**TSGf18423f**	4	12.9	4		1.1
**TSGf18423f**	4		4.2		1.1
**TSGf18423f**	3.7		3.8		0.9
**TSGf18423f**	4.5		4.4		1.3
**TSGf18423f**	3.6		3.8		1
**TSGf18423f**	3.8	12	3.8		0.9
**TSGf18423f**	3.4		3.5		0.7
**TSGf18423f**			3.4		
**TSGf18423f**			3.3		
**TSGf18423f**			3.8		1
**TSGf18423f**			3.3		
**TSGf18423f**			3.3		
**TSGf18420b**	4.7	7.1	4.7		1
**TSGf18420b**	5.5	7	4.6		
**TSGf18420b**	4.6	9	4.8		1.2
**TSGf18420b**	5.1		5.2		
**TSGf18420b**			5.4		

**TSGf18423g**	3.9	7	5.2	52	1.5
**TSGf18423g**	4.3		4.5		1.8
**TSGf18423g**	4.2	10	4.4		
**TSGf18423g**	5		4.8		
**TSGf18423g**			5.2		
**D14-N1-2-02**	4.6	8.7	4.8	137	
**D14-N1-2-02**	4.5		4.8		1.6
**D14-N1-2-02**	5		5.2		1.9
**D14-N1-2-02**	4.8		5		
**D14-N1-2-02**			4.9		1.5
**D14-N1-2-02**			4.7		
**D14-N1-2-02**			3.9		1
**D14-N1-2-02**			4		
**D14-N1-2-02**			4.2		1.1
**D14-N1-2-02**			3.9		
**D14-N1-2-02**			4.4		
**D14-N1-2-02**			3.8		
**D14-N1-2-02**			5		1.5
**D14-N1-2-02**			4.2		
**D14-N1-2-02**			4		
**D14-N1-2-02**			3.9		
**D14-N1-2-02**			4		
**TSGf18424**	2.8	7.5	3	84	
**TSGf18424**	3		3.1		
**TSGf18424**	2.7		3		
**TSGf18424**	3.8		4		1.2
**TSGf18424**	2.8		3.7		
**TSGf18424**	3.2	8	4.3		1.2
**TSGf18424**	3.2		3.8		0.9
**TSGf18424**	4.2	14	4		1
**TSGf18424**	3.9	11	4.1		1.1
**TSGf18424**			3.1		
**TSGf18424**			3.2		
**TSGf18424**			3.8		1.3
**TSGf18424**			3		
**TSGf18424**			3.1		
**TSGf18424**			2.8		
**TSGf18424**			3.2		
**TSGf18424**			2.9		
**TSGf18424**			2.7		
**TSGf18424**			3.2		
**TSGf18424**			3.2		
**TSGf18424**			2.9		
**TSGf18424**			3.5		
**TSGf18425a**	5.4	22	6.1	327	2.2
**TSGf18425a**	6.2	23.5	6.1		2.1

**TSGf18425a**	5.3	20	5.4		1.9
**TSGf18425a**	5.8		6		2.1
**TSGf18425a**	5.5	19	5.8		1.9
**TSGf18425a**	5.1	14	5.9		
**TSGf18425a**			6.5		2.3
**TSGf18425a**			6.1		2.2
**TSGf18425a**			6.7		2.4
**TSGf18425a**			5.8		1.7
**TSGf18425a**			6		2
**TSGf18425b**	5.8		6.1	212	2.2
**TSGf18425b**	5.5	19	5.8		2
**TSGf18425b**	5.3	21	5.5		1.8
**TSGf18425b**	6		6.1		2
**TSGf18425b**	5		5.8		1.7
**TSGf18425b**	5.5	18.5	5.7		1.8
**TSGf18425b**	5.8		6		2.2
**TSGf18425b**	5.8	21.5	5.9		2
**TSGf18425b**	6	22	6		2.3
**TSGf18425b**	5.4		5.1		2
**TSGf18425b**	5.5	19.5	6.1		2.2
**TSGf18425b**	6.1		5.5		1.8
**TSGf18425b**			5.7		1.8
**TSGf18425b**			6.2		1.9
**TSGf18425b**			6.6		2.4
**TSGf18425b**			6		2
**TSGf18425b**			5.8		1.9
**TSGf18425b**			6.2		
**TSGf18425b**			6.3		2.3
**TSGf18425b**			5.9		2
**TSGf18425b**			5.4		1.6
**TSGf18425b**			5.8		1.9
**TSGf18425b**			6.3		2.3
**TSGf18425c**	5.7	15.5	6.1		
**TSGf18425c**	5.7		6.2		
**TSGf18425c**	6	20	6.4		2.3
**TSGf18425c**	5.6		5.9		2.1
**TSGf18422**	6.2		6.5	210	2
**D14-N1-2-001**	5.2		5.4	200	
**D16-HA-74-2-01**	6.1				
**D16-HA-74-2-01**	5		5.9		1.9
**D16-HA-74-2-01**	5.8		6.1		2
**D16-HA-74-2-01**	6	20.5	6.3		2.1
**D16-HA-74-2-01**	5.4		5.9		
**D16-HA-74-2-01**	5.7		6.2		2.2
**D16-HA-74-2-01**	5.5		6		

**D16-HA-74-2-01**	5.7	19.5	6.1		2
**D16-HA-74-2-01**			5.8		
**D16-HA-74-2-01**			5.7		1.9
**D16-HA-74-2-01**			5.5		1.7
**D16-HA-74-2-01**			6		2.1
**D16-HA-74-2-01**			5.2		
**D16-HA-74-2-01**			5.5		
**D16-HA-74-2-01**			5.5		
**Mean measurements of all specimens**	5.02	15.5	5.05	161.48	1.59
**Standard deviation**	0.93	5.37	1.02	84.55	0.52

Supplementary Table 1. Measurements of* Cyathinema digermulense *gen. et sp. nov. fossils and their morphological elements*. *(a) *A table showing measured values for mean width and lengths and standard deviation of the tubular threads, and mean diameter and standard deviation of the cup-shaped structures per individual* Cyathinema *specimen in macerates and thin sections.* ***(b)*** *All measurements of the morphological elements of* Cyathinema *in the studied material, including width and length of the tubular threads, diameter of the cup-shaped structures, overall sheet size, diameter of the inner opening, and standard deviation. These data are presented as a graphic in the main text (Fig. 4). Specimens not given a museum number under prefix TSGf were destroyed during Raman spectroscopy analysis.*

**Characteristic features of *C. digermulense***	**red algae (Rhodophyta)**		**Chanofla-gellata**	**Mesomycetozoea, Opisthokonta (slime moulds)**	**Demospongiae, Porifera**	**Placozoa**	**Nematophyta (polyphyletic)**
	**Florideophyceae**	**Corallinales**					
**tubular elements**	+	+	+	+	+	+	+
**stacked tubular treads**	+	+	+	+	+	+	+
**reticulated layer**	+	+			+	+	+
**cup-shaped structure**	+	+	?	+	+		+
**inner opening**	+	+	+				
**apical-basal axis**	+	+	+	+	+	+	+
**preservation potential**	+	+			+	?	+
**Precambrian fossil record**	+	?			?	?	
**Precambrian origin of the clade**	+	?	+	+	+	?	

***Supplementary Table 2. Distribution of morphological characters recognized in* Cyathinema digermulense *gen. et sp. nov. among fossil and extant clades*.** Cyathinema *shares the most characteristics with modern and fossil red algae. In addition to the presence of red algal fossils in contemporaneous strata, this makes the division Rhodophyta the most likely candidate for the affinity of* Cyathinema. *The last category—"Precambrian origin of the clade" is based on molecular clock*

estimates from literature. An epithelial layer is present in a variety of eukaryotic clades, so affinity to other organisms cannot be properly excluded.

Cyathinema digermulense specimen #	Microscopy slide #	Rock sample horizon	Depth below the Mortensens Fm. Diamictite (m)
**TSGf18425a**	**1**	**D16-HA-69**	**0.05**
**TSGf18425b**	**1**	**D16-HA-69**	**0.05**
**TSGf18425c**	**1**	**D16-HA-69**	**0.05**
**TSGf1840a**	**5**	**D14-N1**	**0.2**
**TSGf1840b**	**5**	**D14-N1**	**0.2**
**TSGf1840c**	**5**	**D14-N1**	**0.2**
**TSGf1840d**	**5**	**D14-N1**	**0.2**
**TSGf1840e**	**5**	**D14-N1**	**0.2**
**TSGf18421**	**9**	**D14-N1**	**0.2**
**TSGf18422**	**4**	**D14-N1**	**0.2**
**TSGf18423a**	**6**	**D14-N1**	**0.2**
**TSGf18423b**	**6**	**D14-N1**	**0.2**
**TSGf18423c**	**6**	**D14-N1**	**0.2**
**TSGf18423d**	**6**	**D14-N1**	**0.2**
**TSGf18423e**	**6**	**D14-N1**	**0.2**
**TSGf18423f**	**6**	**D14-N1**	**0.2**
**TSGf18423g**	**6**	**D14-N1**	**0.2**
**D14-N1-2-01**	**2**	**D14-N1**	**0.2**
**D14-N1-2-02**	**2**	**D14-N1**	**0.2**
**TSGf18427a**	**2**	**D14-N1**	**0.02**
**TSGf18427b**	**TS2**	**D14-N1**	**0.02**
**TSGf18424**	**TS1**	**D14-N2**	**2**
**TSGf18426a**	**TS1**	**D14-N2**	**2**
**TSGf18426b**	**TS1**	**D14-N2**	**2**
**D16-HA-74-2-01**	**2**	**D16-HA-74**	**8**

Supplementary Table 3. Occurrence of* Cyathinema digermulense *gen. et sp. nov. specimens per sample, in distance below the Mortensnes Formation diamictite. *Specimens not given a museum number under prefix TSGf were destroyed during Raman spectroscopy analysis.*

References in the Supplementary Information:

1. Schopf, J. W., Kudryavtsev, A. B., Agresti, D. G., Czaja, A. D. & Wdowiak, T. J. Raman Imagery: A new approach to assess the geochemical maturity and biogenicity of permineralized Precambrian Fossils. **5**, 333–371 (2015).
2. Lahfid, A. *et al.* Evolution of the Raman spectrum of carbonaceous material in low-grade metasediments of the Glarus Alps (Switzerland). *Terra Nov.* **22**, 354–360 (2010).
3. Russell, G. & Veltkamp, C. J. Epiphyte survival on skin-shedding macrophytes. *Mar. Ecol. Ser.* **18**, 149–153 (1984).

4. Maldonado, M. The ecology of the sponge larva. *Can. J. Zool.* **84**, 175–194 (2006).
5. Adl, S. M. *et al.* Revisions to the Classification, Nomenclature, and Diversity of Eukaryotes. *J. Eukaryot. Microbiol.* **66**, 4–119 (2019).
6. Knoll, A. H. The Multiple Origins of Complex Multicellularity. *Annu. Rev. Earth Planet. Sci.* **39**, 217–239 (2011).
